# Molecular dynamics simulations reveal key roles of the LIF receptor in the assembly of human LIF signaling complex

**DOI:** 10.1016/j.csbj.2025.01.014

**Published:** 2025-01-27

**Authors:** Bo Gao, Hanrui Liu, Mengkai Zhu, Shun Zhang, Meiniang Wang, Yijun Ruan, Yue Zheng

**Affiliations:** a Life Sciences Institute, Zhejiang University, Hangzhou, Zhejiang, China; b BGI Research, Hangzhou 310030, China; c BGI Research, Changzhou 213299, China

**Keywords:** Molecular Dynamics, LIF, Gp130, LIFR

## Abstract

Leukemia inhibitory factor (LIF) is a critical cytokine involved in various biological processes, including stem cell self-renewal, inflammation, and cancer progression. Structural studies have revealed how LIF forms a functional signaling complex. However, the dynamic binding pattern of the complex remains inadequately clarified. In this study, we employed molecular dynamics (MD) simulations to investigate the recognition and binding mechanisms of LIF, revealing a preferential affinity for LIF Receptor (LIFR) over gp130, attributable to a larger buried surface area at the LIF–LIFR interface. Key residues F178 and K181 in FXXK motif, along with K124 in LIF helix B, mediate hydrophobic interactions, hydrogen bonding and allosteric regulation, collectively stabilizing the LIF-LIFR interaction. We propose that the unique N-terminal extension of LIF enables signaling without requiring the additional receptor subunit beyond gp130 and LIFR, as verified by cell proliferation assays, distinguishing it from other cytokines in the LIF family. Additionally, analysis of domain fluctuations revealed that the LIF–LIFR interface undergoes less angular displacement compared to the LIF–gp130 interface, indicating a more stable interaction with LIFR. Together, these findings provide valuable insights into the molecular basis of LIF recognition and binding, offering a dynamic foundation for cytokine engineering.

## Highlights

• Larger buried surface area at LIF–LIFR vs. LIF–gp130 drives preferential binding of LIF to LIFR.
• Key residues K124, F178, K181 mediate allostery, hydrophobic and H-bond interactions, stabilizing LIF-LIFR binding
• LIF’s unique N-terminal extension enables signaling without additional receptor subunits beyond gp130 and LIFR.
• LIF–LIFR interface shows less angular displacement than LIF–gp130 indicating a more stable interaction.

## Graphical Abstract

**Figure:**
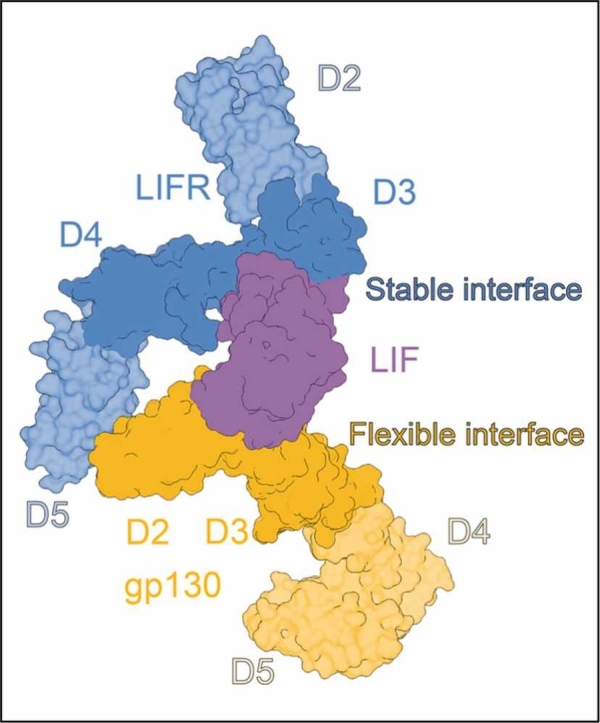

## 1 Introduction

Leukemia inhibitory factor is a pleiotropic and significant cytokine involved in a wide variety of activities such as regulating the growth and differentiation of leukemia and hematopoietic cells, stem cell self-renewal, embryo implantation, inflammatory responses, and cancer progression[1], [2], [3], [4], [5], [6], [7], [8], [9]. LIF is also a member of the Interleukin-6 cytokine family, which includes IL-6, IL-11, LIF, ciliary neurotrophic factor (CNTF), oncostatin M (OSM), cardiotrophin-1 (CT-1), and cardiotrophin-like cytokine factor 1 (CLCF1)[10], [11], [12], [13], [14], [15], [16], [17], [18]. Initially, LIF was identified as a factor that inhibits proliferation of mouse myeloid leukemia M1 cells[13]. Subsequent studies have shown that LIF can also promote the proliferation of leukemia cells and various other cell types[3], [7], [13], [19], [20], [21], [22]. Moreover, LIF is essential for maintaining the self-renewal and pluripotency of embryonic and neutral stem cells [4], [5], [23], [24].

As classic four-helix bundle proteins, cytokines in the IL-6 family share a universal signal transducing receptor, gp130[25], [26]. Unlike IL-6, which forms a homodimer signaling complex with gp130 and the IL-6 receptor α subunit (IL-6 Rα)[27], LIF assembles a 1:1:1 tripartite complex with gp130 and the different chaperone LIF receptor[28]. Similarly, CNTF and CLCF1 also require gp130-LIFR for signal transduction; however, unlike LIF, they first bind to CNTFα before assembling into a 1:1:1:1 quaternary signaling complex with gp130 and LIFR to initiate signaling [29]. Structural differences among these complexes result in functional diversity within the cytokine family, influencing cell fate in various tissues. Therefore, elucidating the recognition and binding mechanism of LIF is essential for understanding its signal transduction mechanism.

Although several research groups have determined the structures of LIF using NMR[30], X-ray[25], [31], [32], and Cryo-EM[29], the dynamic binding behavior of LIF remains poorly understood. This gap arises from the inherent limitations of these structural biology techniques in capturing the conformational diversity of cytokine dynamics. Notably, dynamic allosteric activation of latent TGF-β signaling has been reported[33], with valuable dynamic structural information on TGF-β obtained through extensive 3D classification analyses. For biomacromolecules with diverse conformations, such as the LIF complex, molecular dynamics (MD) simulations could offer additional insights into their mechanisms of action and may elucidate the sequential binding of LIF to the LIFR and gp130 receptors.

In this study, we conducted molecular dynamics simulations of LIF and its core complex on a near-microsecond (μs) timescale. By comparing the buried surface areas of the interfaces, we observed that LIF preferentially binds to LIFR over gp130, as evidenced by the larger buried surface area at the LIF-LIFR interface compared to the LIF-gp130 interface. Monitoring the distances at key sites and calculating hydrogen bond frequency revealed that key residues F178 and K181 within the FXXK motif, along with K124 in LIF helix B, mediate hydrophobic interactions, hydrogen bonding, and allosteric regulation, playing a pivotal role in stabilizing the LIF–LIFR interaction. Based on structural comparison and cell proliferation experiments, we propose that the unique N-terminal extension of LIF enables signaling without requiring an additional receptor subunit beyond gp130 and LIFR, distinguishing it from other cytokines in the LIF family. Our analysis of domain fluctuations revealed that the angle between LIF and gp130 (52–62°) was consistently larger than the angle between LIF and LIFR (55–59°) during the MD simulations. Taken together, our findings provide a deeper mechanistic understanding of LIF binding pattern and offer structural insights into cytokine dynamics.

## 2 Results

### 2.1 Molecular dynamic simulations of LIF signaling core complex

**Fig. 1:**
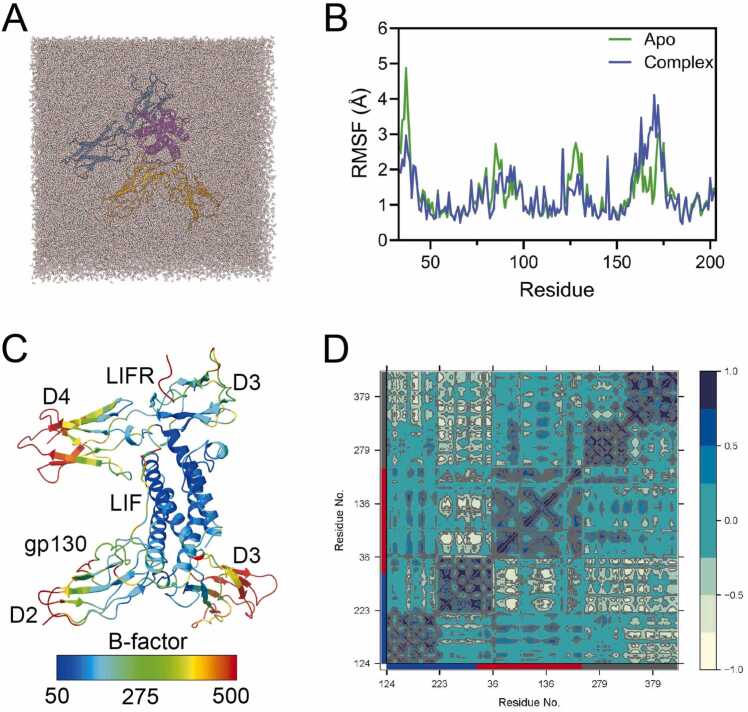
Overall the LIF core complex MD simulation. (A) Snapshot of molecular dynamics simulation of the LIF core complex solution system. Water molecules are shown as red and white sticks, and the LIF structure is depicted in cartoon style with a purple color, gp130 is colored in goldenrod, and LIFR is displayed in blue. (B) RMSF of LIF in the LIF and LIF core complex MD simulations. The green line represents the apo system, and the blue line represents the complex system, (C) The LIF core complex structure are colored according to the B-factor during MD simulation. Higher B-factor values correspond to larger fluctuations. (D) Correlation map of the MD simulation for the LIF core complex. The blue box represents the gp130 residue range, the red box labels the LIF residue range, and the gray box represents the LIFR residue range.

To elucidate the recognition and binding mechanisms underlying LIF signal transduction, we performed MD simulations of LIF and, for computational efficiency, its core complex, focusing on the interaction interfaces between the D2 and D3 domains of gp130, LIF, and the D3 and D4 domains of LIFR. The structures of LIF and its core complex were prepared using the CHARMM36m force field[34] within GROMACS[35]. Following solvation, ion addition, energy minimization, and pressure equilibration, we conducted three independent molecular dynamics simulations for each system: 1000 ns for LIF and 500 ns for the complex (Fig. 1A; Figs. S1–3, Video S1 and S2, Supporting Information).

Supplementary material related to this article can be found online at doi:10.1016/j.csbj.2025.01.014.

The following is the Supplementary material related to this article Video S1 and Video S2.

Video S1 LIF MD simulation over 1 μs. One frame was captured every 2 ns. LIF is shown in cartoon style with rainbow color scheme.

Video S2 LIF core complex MD simulation over 500 ns. One frame was captured every 1 ns. LIF is shown as purple color, gp130 is colored in goldenrod, and LIFR is displayed in blue.

Upon comparing the root mean square fluctuation (RMSF) of LIF in its apo form with that in the complex, we found that the residue-wise trends were generally similar, except in the N-terminal, AB loop, and BC loop regions, where the RMSF values were significantly higher in the apo form than in the complex (Fig. 1B). Calculating the overall B-factor during the complex MD simulations revealed that the classic cytokine core four-helix bundle was highly stable, and the regions involved in the LIF-LIFR and LIF-gp130 interfaces exhibited reduced flexibility (Fig. 1C). In contrast, the outer regions of the D3 domain of gp130, which do not participate in recognition and binding, exhibited high flexibility. Similarly, the outer regions of the D4 domain of LIFR demonstrated comparable flexibility. This trend was also reflected in the RMSF curves of both gp130 and LIFR (Fig. S4, Supporting Information).

Upon examining the atom-wise cross-correlation coefficients (Fig. 1D), we observed a slight positive correlation between the D2 domain of gp130 and LIF, excluding internal motions within each subunit. However, the majority of the D3 domain of gp130 showed significant negatively correlated motion with LIF and LIFR. In contrast, LIF generally moved in concert with both the D3 and D4 domains of LIFR.

### 2.2 Interaction dynamics features of LIF-LIFR

**Fig. 2:**
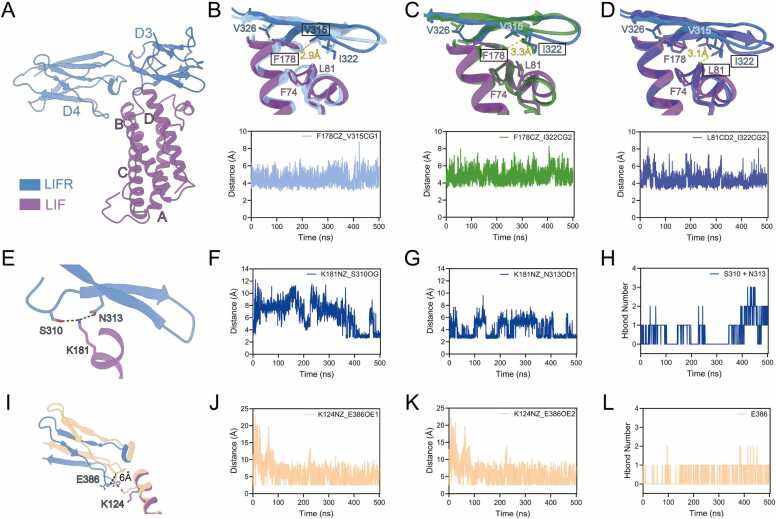
Interaction dynamic Features of LIF-LIFR. (A) The overall cartoon view of LIF binding to LIFR. LIF is shown in purple, LIFR is displayed in blue. (B) The representative structure of the F178-V315 hydrophobic interaction is shown in sky-blue color, and the cryo-EM structure colored as in A. The chart below shows the distance between the CZ atom of F178 in LIF and the CG1 atom of V315 in LIFR during the MD simulation. (C) The representative structure of the F178-I322 hydrophobic interaction is shown in forest green color, and the cryo-EM structure colored as in A. The chart below shows the distance between the CZ atom of F178 in LIF and the CG2 atom of I322 in LIFR during the MD simulation. (D) The representative structure of the L81-I322 hydrophobic interaction is shown in royal blue color, and the cryo-EM structure colored as in A. The chart below shows the distance between the CD2 atom of L81 in LIF and the CG2 atom of I322 in LIFR during the MD simulation. (E) The representative structure showing K181 forming hydrogen bonds with S310 and N313. Colors as in A. (F) The distance between the NZ atom of K181 in LIF and the OG atom of S310 in LIFR during the MD simulation. (G) The distance between the NZ atom of K181 in LIF and the OD1 atom of N313 in LIFR during the MD simulation. (H) K181 forms hydrogen bonds with S310 and N313 during the MD simulation. (I) Zoom structural view showing K124 undergoing an upward flip, positioned to interaction with E386. The loop in the D4 domain displays a 6 Å inward displacement relative to LIF. The representative structure of the K124-E386 electrostatic interaction and hydrogen bond formation is shown in vanilla-colored cartoon style, while the cryo-EM structure colored as A. (J) The distance between the NZ atom of K124 in LIF and the OE1 atom of E386 in LIFR during the MD simulation. (K) The distance between the NZ atom of K124 in LIF and the OE2 atom of E386 in LIFR during the MD simulation. (L) K124 forms hydrogen bonds with E386 during the MD simulation.

The four helices of LIF bind to LIFR in a manner analogous to “chopsticks” (Fig. 2A), with the FXXK motif in LIF playing a critical role in the LIF-LIFR interaction[36]. The most striking feature of the interaction dynamics is that F178 binds into a groove composed of the D3 domain of LIFR and the AB loop turn of LIF (Fig. S5, Supporting Information). Specifically, F178 inserts into a hydrophobic pocket formed by LIFR residues V315, I322, V326, and LIF residues F74, L81. By monitoring the distances between the CZ atom of F178 in LIF and the CG1 atom of V315 and the CG2 atom of I322 in LIFR, we observed that these atoms frequently remain in close proximity, within the range of hydrophobic interaction (Fig. 2B and C). Interestingly, residues F74 and L81 in the AB loop turn form another half of the pocket. The distance between the CD2 atom of L81 in LIF and the CG2 atom of I322 in LIFR is maintained at 4 Å or below (Fig. 2D). Although the RMSF of the AB loop turn (residues 70–82) does not show a significant difference (Fig. S6, Supporting Information), by classifying their secondary structure characteristics, it is clear that the helix turn represented by F74 and L81 is more stable in the MD simulations of the complex due to the presence of the β-sheet in LIFR (Fig. S7, Supporting Information).

Another representative site, K181, forms hydrogen bonds with both S310 and N313 (Fig. 2E). Measuring the distance between the NZ atom of K181 in LIF and the OG atom of S310, as well as the OD1 atom of N313, revealed that their distances are sometimes less than 3 Å (Fig. 2F and G). During the simulation, the number of hydrogen bonds correlates with the distance between these atoms (Fig. 2H). Previous cryo-EM structure indicate that K181 forms a hydrogen bond exclusively with S310, while the crystal structure of human LIF-mouse LIFR complex indicates that both residues S262 and N265[32], which are conserved in human LIFR sequences, form hydrogen bonds. However, across three independent molecular dynamics simulations, the formation of hydrogen bond involving residue N313 is significantly more frequent than that involving S310 (Fig. S8 A-C, Supporting Information). We propose that N313, located on the β-hairpin motif of LIFR, is more likely to interact with K181 due to the hydrophobic interactions between V177 in LIF and V326 and F328 in LIFR, as observed in the MD simulations (Fig. S8 D-E, Supporting Information). Additionally, F178 interacts with the hydrophobic pocket formed by the β-strands (Fig. S5, Supporting Information), where N313 resides, thereby promoting the interaction between K181 in LIF and N313 in LIFR. In contrast, S310 is positioned on a long, flexible loop. The hydrophobic interactions mediated by V177 and F178, along with the hydrogen bond between K181 and N313, draw the loop closer to K181, facilitating the formation of hydrogen bonds between K181 in LIF and S310 in LIFR. These interactions collectively contribute to the stabilization of the LIF–LIFR interface.

Our MD study elucidates the structural basis of the interaction between LIF and the D4 domain of LIFR, with K124-E386 mediating binding recognition. Compared to the cryo-EM structure, the side chain of K124 in LIF helix B undergoes an upward flip, accompanied by a 6 Å inward displacement of the loop in the D4 domain relative to LIF (Fig. 2I; Fig. S9A, Supporting Information). This conformational allostery facilitates electrostatic interactions and hydrogen bond formation (Fig. 2M-O). Analysis of the hydrogen bond formation frequency across the MD simulations revealed an average occurrence of approximately 40 % over three independent runs (Fig. S9B, Supporting Information). In alignment with our dynamic structural findings, a previous alanine substitution study demonstrated that K124 plays a significant role in LIFR binding, with its substitution resulting in a twofold reduction in binding affinity[37]. Sequence alignment reveals that K124 is highly conserved within the mammalian LIF family (Fig. S10, Supporting Information), but not in other cytokines that also interact with LIFR.

### 2.3 Interaction dynamics features of LIF-gp130

**Fig. 3:**
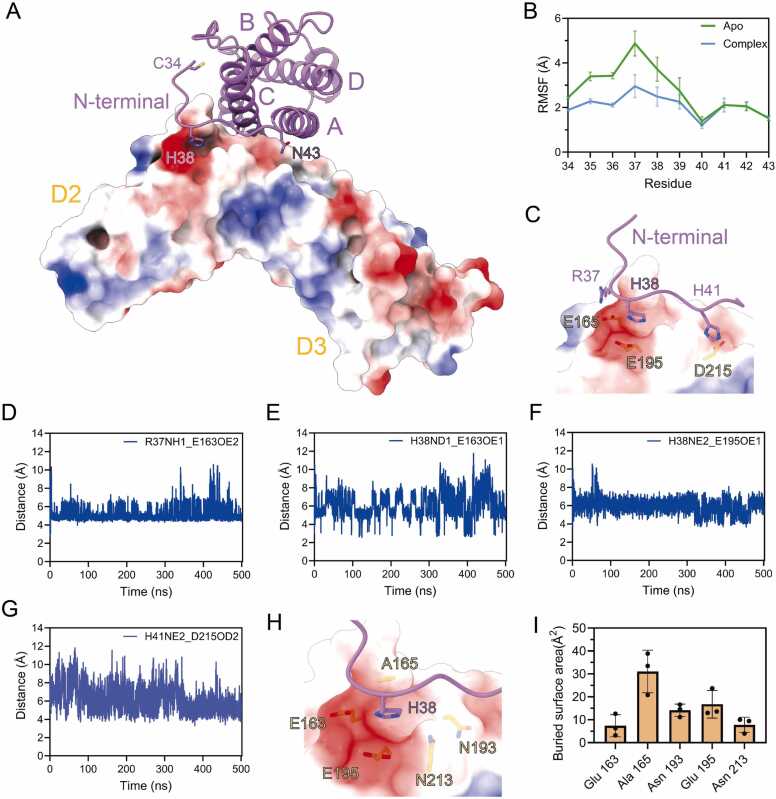
Interaction dynamic Features of LIF-gp130. (A) The overall view of LIF binding to gp130. LIF is shown in purple cartoon style, gp130 surface is colored according to the electrostatic surface potential (blue, +5 kT; red, −5 kT). (B) RMSF of the N-terminal extension in the LIF and LIF core complex MD simulation. The green line represents the apo system, and the blue line represents the complex system. Error bars represent mean ± SD of n = 3 experiments. (C) A patch of positively charged residues of LIF are positioned to make electrostatic interactions with negatively charged region in the gp130 subunit. Contact residues are shown as sticks. gp130 surface is colored according to the electrostatic surface potential (blue, +5 kT; red, −5 kT). (D-G) Distances monitored during the MD simulation between key LIF residues and their corresponding gp130 residues. (D) R37 (NH1) in LIF and E163 (OE2) in gp130. (E) H38 (ND1) in LIF and E163 (OE1) in gp130. (F) H38 (NE2) in LIF and E195 (OE1) in gp130. (G) H41 (NE2) in LIF and D215 (OD2) in gp130. (H) H38 inserts into the negatively charged pocket of gp130. Contact residues are shown as sticks. Colors as in C. (I) The representative residues forming the negatively charged pocket of gp130 that interacts with H38 during the MD simulation. Error bars represent mean ± SD of n = 3 experiments.

The recognition mechanism of LIF-gp130 is predominantly mediated by the N-terminal extension of LIF, which acts as a “door latch” by inserting into the negatively charged pocket of gp130 (Fig. 3A). Owing to electrostatic interactions with gp130, the N-terminal extension of LIF exhibits significantly reduced RMSF in the LIF core complex compared to LIF alone (Fig. 3B). Residues R37 and H38 predominantly engage with negatively charged region on gp130, notably E163 and E195, as evidenced by consistently close side-chain atom distances (Fig. 3C-F), contributing significantly to the buried surface area. Additionally, the NE2 atom of H41 establishes a significant interaction with the OD2 atom of D215 throughout the MD simulations (Fig. 3C and G). Subsequent solvent-accessible surface areas (SASA) analysis across three independent MD simulations identified key residues in gp130 involved in van der Waals interactions with R37, H38, and H41. Specifically, H38 interacts with E163, A165, N193, E195, and N213 of gp130; R37 engages with E163, A165, T166, and E195; while H41 contacts with F191, N193, and D215 (Fig. 3H-I; Fig. S11 and S12, Supporting Information).

### 2.4 N-terminal extension is critical for complex binding in LIF cytokine family

**Fig. 4:**
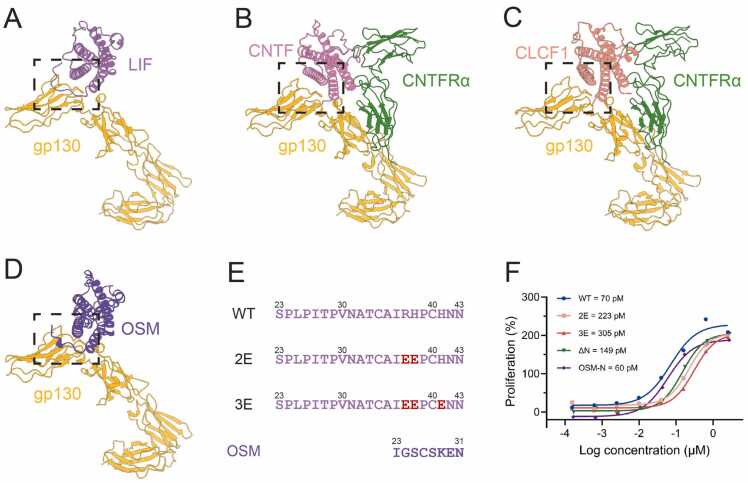
N-terminal extension is critical for complex binding in LIF cytokine family. (A) The cartoon representation of LIF–gp130, with LIF in purple and gp130 in goldenrod. The dotted box highlights the N-terminal extension of LIF. (B) The cartoon representation of CNTF-gp130-CNTFRα (PDB: 8D74), with CNTF in hot pink, gp130 in goldenrod and CNTFRα in forest green. The dotted box highlights the N-terminus of CNTF. (C) The cartoon representation of CLCF1-gp130-CNTFRα (PDB: 8D7R), with CLCF1 in light coral, gp130 in goldenrod and CNTFRα in forest green. The dotted box highlights the N-terminus of CLCF1. (D) The cartoon representation of OSM-gp130, with OSM in blue violet and gp130 in goldenrod. The dotted box highlights the N-terminal region of OSM. The structure is predicted by AlphaFold. (E) The sequences of the N-terminal for LIF and its variants: wild-type (WT), 2E (R37E and H38E), 3E (R37E, H38E and H41E), and the chimeric OSM N-terminal. (F) TF-1 cell proliferation activity induced by LIF and its variants.

Among the cytokine family that utilizes LIFR, only LIF possesses a long N-terminal extension, whereas OSM has only a small segment, and CNTF and CLCF1 lack any extension (Fig. 4A-D). Structural comparisons indicate that CNTF and CLCF1 rely on the CNTF α subunit to form a complex assembly necessary for signal transduction. The N-terminal extension of LIF functions as a “latch”, with three basic amino acids mediating interactions with the negatively charged regions of gp130, primarily through the insertion of the H38 side chain into a negatively charged pocket. Furthermore, charge reversal substitution at two or three of LIF residues (R37, H38 and H41) results in a three- and four-fold decrease in EC50, respectively, as measured by TF-1 cell proliferation assays (Fig. 4 E and F; Fig. S13, Supporting Information). Truncation of the N-terminal region leads to a one-fold reduction in its proliferation activity. However, transplantation of the active OSM N-terminal onto the LIF N-terminal restored proliferation activity to wild-type levels, likely due to the reintroduction of elements from the N-terminal extension and the basic amino acid.

### 2.5 Comparison of LIF-LIFR and LIF-gp130 Interfaces

**Fig. 5:**
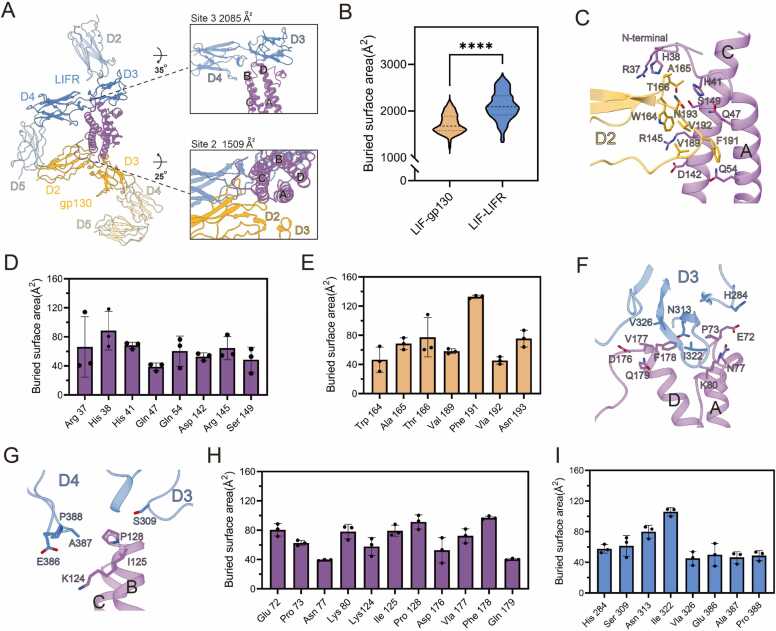
Comparison of LIF-LIFR and LIF-gp130 Interfaces. (A) The overall and zoom cartoon view of the LIF complex. LIF is shown in purple, LIFR is displayed in blue, and gp130 is colored in goldenrod. The rest of the structure, outside the core complex, is displayed as transparent. Above the zoom structural view, the average buried surface area corresponding to this interface is displayed. (B) The representative buried surface area of LIF-LIFR and LIF-gp130 Interfaces. **** , p < 0.0001. (C) The structural view of LIF-gp130 Interfaces. Contact residues during the MD simulation are shown as sticks. (D) Histograms of buried surface area for LIF residues at Site 2. Error bars represent mean ± SD of n = 3 experiments. (E) Histograms of buried surface area for gp130 residues at Site 2. Error bars represent mean ± SD of n = 3 experiments. (F) The structural view of the interaction between helices A and D of LIF and the D3 domain of LIFR. Contact residues during the MD simulation are shown as sticks. (G) The structural view of the interaction between helices B and C of LIF and the D3 and D4 domains of LIFR. Contact residues during the MD simulation are shown as sticks. (H) Histograms of buried surface area for LIF residues at Site 3. Error bars represent mean ± SD of n = 3 experiments. (I) Histograms of buried surface area for LIFR residues at Site 3. Error bars represent mean ± SD of n = 3 experiments.

While several structural studies have elucidated the binding mechanism of the LIF complex, the molecular basis underlying the significantly higher affinity of LIF for LIFR compared to gp130 remains inadequately characterized. Notably, the cryo-EM complex structure reveals no substantial difference between the two interfaces (LIF-gp130: 1412 Å², LIF-LIFR: 1439 Å²)[29]. To investigate this further, we calculated the solvent-accessible surface areas of LIF, LIFR, gp130, and their respective LIF-LIFR and LIF-gp130 complexes at each nanosecond to assess the buried surface area. The analysis indicates that the average buried surface area of the LIF-LIFR interface, derived from three independent MD simulations, is over 500 Å² larger than that of the LIF-gp130 interface (Fig. 5A and B; Fig. S14, Supporting Information), with a statistically significant difference. Moreover, the number of representative residues at the LIF-gp130 interface, which contribute to a surface area exceeding 38 Å² with another chain, is significantly smaller compared to the LIF-LIFR interface. These residues are primarily localized to the D2 domain of gp130, where they interact with LIF helices A and C, as well as the N-terminal loop (Fig. 5C-E). Specifically, R37, H38, and H41 from the LIF N-terminal, along with Q47 and Q54 from helix A, and R145 and S149 from helix C, engage with W164, A165, T166, V189, F191, V192, and N193 of the gp130 D2 domain.

In contrast, the characteristic contact residues of the LIF-LIFR interface encompass not only the D3 and D4 domains of LIFR, but also LIF helices B and D, as well as several loop regions, including the AB, BC and CD loops (Fig. 5F-I). Specifically, E72, P73, N77, and K80 on the AB loop, D176 on the CD loop, and V177, F178, and Q179 on helix D maintain consistent interaction with the β-sheet structure of the LIFR D3 domain, particularly at residues H284, N313, I322, and V326. Furthermore, at the adjacent interface, P128 on the BC loop, along with K124 and I125 from helix B, interacts with residue S309 of the LIFR D3 domain and residues E386, A387, and P388 of the D4 domain.

### 2.6 Dynamics analysis of LIF-gp130-LIFR core complex

**Fig. 6:**
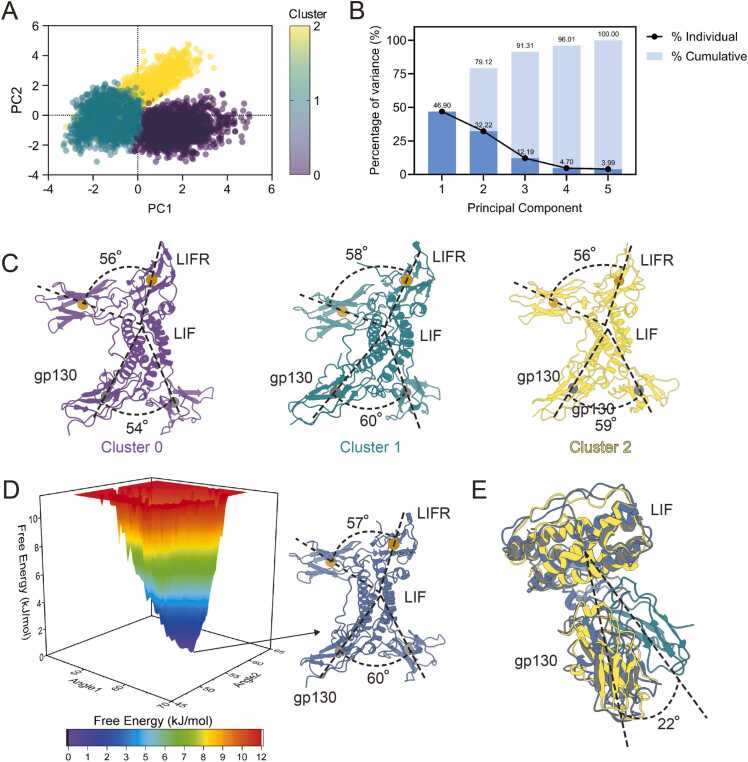
Dynamics analysis of gp130- LIF -LIFR core complex. (A) Principal component analysis (PCA) scores of the dataset. Cluster 0 is shown in purple, Cluster 1 in teal, and Cluster 2 in yellow. (B) Percentage of variance explained by each principal component in the PCA. The individual percentage is represented by the black line and spheres, indicating the variance explained by each individual principal component. The cumulative percentage is shown by the sky-blue columns, representing the total variance explained by the combined principal components. (C) Angles between gp130 and LIFR relative to LIF in the representative structures of each cluster. Colors as in A. (D) The three-dimensional Gibbs energy landscape of MD domain fluctuation angles. Angle1 represents the angle between LIF and the COM (center of mass) of the gp130 D2 and D3, Angle2 represents the angle between LIF and the COM of the LIFR D3 and D4. The structure of Gibbs free energy landscape minimum is colored as gray-blue. (E) Superposition of the representative structures of Cluster 1 and Cluster 2, the cryo-EM structure, and the structure corresponding to the minimum of the Gibbs free energy landscape. Cluster 1 and Cluster 2 are colored as in A, the cryo-EM structure is shown in gray, and the structure corresponding to the minimum of the Gibbs free energy landscape is displayed in gray-blue.

To further analyze the recognition and binding mechanism, several parameters were calculated, including the angle between LIF and the center of mass (COM) of the gp130 D2 and D3 domains, the angle between LIF and the COM of the LIFR D3 and D4 domains, and the distance between gp130 D2 and LIFR D4 domains, as well as between gp130 D3 and LIFR D3 domains (Fig. S15, Supporting Information). Notably, the angular variation of LIFR was significantly smaller than that of gp130. Additionally, the distances between gp130 D3 and LIFR D4, as well as gp130 D3 and LIFR D3, demonstrated a correlated trend. To further explore these findings, Gaussian Mixture Model was employed to analyze the four features in greater depth. The Elbow classification method indicates that the best Bayesian Information Criterion (BIC) and Akaike Information Criterion (AIC) scores were obtained when the number of classification groups was set to 3 (Fig. S16A, Supporting Information). Principal component analysis (PCA) was subsequently applied, effectively distinguishing the data along PC1 and PC2 axes (Fig. 6A and B; Fig. S16B, Supporting Information). Structural representatives of each cluster center were extracted from the corresponding frames.

The interface between LIF and LIFR remains relatively stable throughout the simulations. In contrast, the D3 domain of gp130 displays rotational movement relative to LIF (Fig. 6C), accompanied by corresponding changes in the distance between gp130 D3 and LIFR D3 domains (cluster 0: 59 Å, cluster 1: 51 Å, cluster 2: 56 Å) (Fig. S17, Supporting Information). Among these, cluster 2 most closely resembles the cryo-EM structure, with an RMSD of 2.0 Å (across all 197 pairs) (Fig. S18, Supporting Information).

Further analysis of angular variations between LIF and the receptor domains in MD simulations reveals that the angular displacement between LIF and gp130 predominantly ranges from 52° to 62°, whereas the angle between LIF and LIFR remains confined between 55° and 59°. To further characterize these interactions, we constructed Gibbs free energy landscapes through Boltzmann inversion of multi-dimensional histograms (Fig. 6D). Analysis of the minimum energy configuration on the Gibbs free energy landscape indicates that the LIF–LIFR angle stabilizes at 57°, whereas the LIF-gp130 angle is 60°, with the distances corresponding to those observed in the representative structure of cluster 2 (Fig. S17 and S19, Supporting Information). Although the angular characteristics of cluster 1 closely resemble those of cryo-EM model and the minimum structure on Gibbs free landscape, structural comparison reveals that gp130 exhibits a 22° rotation relative to LIF in the representative structure of cluster 1 (Fig. 6E), resulting in noticeable differences in the distances between gp130 and LIFR (Fig. S17 and S19, Supporting Information). The structure of Gibbs free energy landscape minimum, derived from Boltzmann inversion, closely aligns with the cryo-EM structure (Fig. S20, Supporting Information). This suggests that the conformation with the highest distribution probability in the MD simulations closely mirrors the most stable conformation in aqueous solution captured by cryo-EM, providing indirect support for cross-validation between the computational and experimental results.

## 3 Discussion

Several structural biology studies have elucidated the four-helix cytokine characteristics of LIF, the architecture of LIF-gp130, LIF-mLIFR, and their complex [25], [29], [31], [32]. However, structural techniques such as X-ray and single-particle cryo-EM provide limited insights into the dynamics of cytokine recognition and binding[38], [39]. A recent innovative study published in *Cell* revealed that conformational entropy redistribution drives the allosteric activation of latent TGF-β by distinguishing conformational changes using more 3D classification groups rather than averaging particles to improve the local resolution of core region[33]. The persistence of uniformity in algorithmic processing of high-resolution density maps can lead to significant loss of information, particularly regarding the dynamic processes of complex recognition and binding. Therefore, focusing on the dynamic changes in LIF binding to gp130 and LIFR offers a more comprehensive explanation for the substantial differences in affinity between LIF-gp130 and LIF-LIFR.

Our MD simulations have provided valuable insights into the recognition and binding patterns of LIF that could not be captured through cryo-EM alone. Notably, analysis of secondary structure dynamics demonstrated that residues F74 and L81 in the AB loop turn helix form a hydrophobic pocket for F178 binding. Importantly, these hydrophobic residues are also commonly conserved in the LIF cytokine family (Fig. S10, Supporting Information). Furthermore, a hydrogen bond selection mechanism involving K181 was identified, enabling LIF to recognize and anchor to LIFR. While the cryo-EM structure indicate that K181 forms a hydrogen bond with S310, our MD simulations indicated that K181 preferentially forms a hydrogen bond with N313 in LIFR, with the frequency of hydrogen bonding being five times higher than that with S310 (Fig. S8, Supporting Information). Moreover, the LIF-LIFR interface exhibits more significant interaction features than the LIF-gp130 interface (Fig. 2, Fig. 3). By comparing the distances between residues F178 and K181 of LIF and their corresponding interaction sites on LIFR with those between residues R37 and H38 of LIF and their interaction sites on gp130 (Fig. 2, Fig. 3), it was observed that the representative features and anchor residues in the LIF-LIFR interface are in closer proximity, suggesting a more stable LIF-LIFR interaction. Additionally, dynamic conformational changes occur during the recognition and binding between LIF and the D4 domain of LIFR. Specifically, the loop of the D4 domain in LIFR shifts, accompanied by electrostatic interactions and hydrogen bond formation between residues LIF K124 and LIFR E386. Consequently, the LIFR D4 domain exhibits lower RMSF and B-factor values relative to the gp130 D3 domain (Fig. 1C and Fig. S4, Supporting Information). Our findings underscore the key role of the LIF receptor in the assembly of the human LIF signaling complex.

To investigate the specificity of gp130 recognition and binding in LIF cytokine family, we combined analyses of cell line proliferation activity with RMSF data in apo and complex MD simulations, emphasizing the role of the N-terminus in mediating LIF-gp130 binding. In comparison to other cytokines such as CLCF1 and CNTF, which lack this feature, the buried surface area of CNTF-gp130 and CLCF1-gp130 is much smaller than that of LIF–gp130. In vivo, these cytokines require the involvement of an additional subunit to form a fully functional signal transduction complex. Therefore, we propose that the unique N-terminal extension of LIF facilitates signaling without the need for an additional receptor subunit beyond gp130 and LIFR, distinguishing it from other cytokines in the LIF family.

In addition to mediating the initial recognition of receptor anchors by LIF, maintaining the binding status is crucial for affinity maturation. By analyzing the differences in the buried surface area of the two interfaces during MD simulations, we observed that the interaction surface of LIF–LIFR is significantly larger than that of LIF–gp130, whereas in the cryo-EM structure, the buried surface areas are nearly identical. Not only is the number of representative snapshots contributing to the buried surface area in LIF–LIFR higher than in the LIF–gp130 interface, but these residues are also distributed across the D3 and D4 domains in LIFR, rather than being confined to the D2 domain of gp130. This suggests that LIF stably binds to both D3 and D4 domains of LIFR, whereas it tends to bind only to the D2 domain of gp130. Due to the combined factors of an increased buried surface area and anchor features, LIF preferentially recognizes and bins to LIFR, subsequently engaging gp130 to facilitate complex formation. A comprehensive analysis of angular variations between LIF and receptor domains in MD simulations reveals that the angular displacement between LIF and gp130 (52°-62°) is larger than that between LIF and LIFR (55°-59°), indicating a more stable LIF-LIFR interface.

LIF exerts several important biological functions, ranging from stimulation of platelets and neuronal formation, hematopoietic stem cells proliferation and acute phase reaction, as well as bone formation[2], [9], [40], [41]. Our study enriches the understanding of the recognition and binding mechanisms of LIF, especially the pattern of the dynamic binding process. The differences in the recognition anchors and the buried surface snapshots trigger the order of LIF recognition and binding in vivo, leading to signal transduction. This not only provides insights into the signaling mechanism of LIF recognition and binding but also offers structural dynamic information of characteristic sites that could aid in further cytokine engineering and support the development of LIF variants with specific function.

## 4 Experimental section

### 4.1 Protein expression and purification

LIF was prepared from *E. coli* strain BL21(DE3) (Invitrogen, Inc) transformed with plasmid pET32a-LIF encoding LIF under the control of the bacteriophage T7 gene promoter. Single colonies of the resulting transformants were used to inoculate 1 l LB broth containing 100 μg/mL ampicillin, cultures were incubated at 37 °C with shaking until OD600 = 0.8, cultures were induced by addition of IPTG to 0.4 mM, and cultures were incubated 16 h at 18 °C. Then cells were harvested by centrifugation (5000 × g; 10 min at 4 °C), resuspended in 75 mL buffer A (50 mM Tris-HCl, pH 8.0, 150 mM NaCl, and 5 % glycerol), and lysed using a homogenizer disrupter (SPXflow, Inc.). The lysate was centrifuged (20,000 × g; 45 min at 4 °C), and the supernatant was loaded onto a 5 mL column of HisTrap HP column (Cytiva, Inc) equilibrated in buffer A and eluted with a 100 mL linear gradient of 0.01–0.5 M imidazole in buffer A. Fractions containing TrxA-LIF were pooled, and Enterokinase (Sangon Biotech, Co) was added to remove the N-terminal tag. The cleaved sample was further purified by cation exchange chromatography with a 100 mL linear gradient of 0.05–1 M NaCl in buffer B (50 mM Tris-HCl, pH 7.5, and 5 % glycerol) to separate the TrxA tag from LIF. The eluate was applied to size exclusion chromatography on a HiLoad 16/600 Superdex 75 column (Cytiva, Inc) equilibrated in buffer C (10 mM Na_2_HPO_4_, 1.8 mM KH_2_PO_4_, PH 7.4, 137 mM NaCl, 2.7 mM KCl). Fractions containing LIF were pooled and stored at −80 °C. Yields were ∼20 mg/L, and purities were > 95 %. The same method was used to purify LIF variants.

### 4.2 Cell proliferation assays

The bioactivity of purified LIF protein and variants were determined following by the Cell Counting-Lite 2.0 Luminescent Cell Viability Assay (Vazyme, Co). Harvested TF-1 cells were resuspended and washed three times with 1640 medium, then transferred into a 96-well flat-bottom plate at approximately 4000 cells/well. LIF was serially diluted 4-fold, with 10 μl of LIF solution added to each well for a final volume of 100 μl. The plate was incubated at 37°C in a humidified atmosphere (5 % CO₂/95 % air). After three days of culture, cell proliferation activity was measured using the Luminescent Cell Viability Assay, according to the manufacturer’s instructions. Absorbance at 560 nm was recorded using a microplate reader (Synergy H1, Co).

### 4.3 Molecular dynamics simulation

Atomistic models of LIF and the LIF core complex were constructed using the PDB entries 1PVH and 8D6A, respectively. The CHARMM36 force field[34] was used to produce the systems. A periodic cubic simulation box with a minimum distance of 1.0 nm between the protein and the box edges was applied. The system was then solvated using the TIP3P water model, and neutralized by adding Na⁺ and Cl⁻ ions to mimic physiological conditions. The system first underwent energy minimization using the steepest descent method. Following this, pre-equilibration was carried out under NVT and NPT conditions, while the temperature was consistently maintained at 310 K. Production of molecular dynamics simulations were conducted with a 2-fs time step, totaling 1000 ns for LIF and 500 ns for the complex. To ensure reproducibility, three independent simulations were performed for each system. Periodic elimination of trajectory, RMSD analysis, RMSF analysis, distance calculation, dictionary of protein secondary structure, extracting the frame’s structure, hydrogen bond analysis, and solvent accessible surface areas analysis were conducted with GROMACS gmx tool[35]. The solution system was examined and analyzed using Visual Molecular Dynamics (VMD 1.94)[42].

### 4.4 B-factor analysis

To represent the fluctuations of the LIF core complex intuitively, B-factor was conducted by GROMACS rmsf tool with the -oq option. The RMSF values were converted to B-factor values and written to a.pdb file. In ChimeraX[43], the LIF core complex structure was colored according to the B-factor values during the MD simulations using the color bfactor command.

### 4.5 Protein dynamic correlation analysis

To reveal the dynamic behavior of the simulated systems, dynamic cross-correlation (DCC) analysis was conducted to evaluate the correlated displacements of the protein backbone atoms (Cα). The cross-correlation matrix (Cij) between residues i and j was calculated using the dccm function from the Bio3D package[44] by R language. This matrix was then used to explore correlated motions across the protein structure, with a focus on Cα atoms, by averaging their displacements from the mean structure. Based on the entire simulation time, the DCC analysis identified regions of the protein that exhibited correlated or anti-correlated motions.

### 4.6 Buried surface area analysis

The buried surface area between LIF and gp130, as well as LIF and LIFR, was calculated using solvent-accessible surface area (SASA) values from the GROMACS gmx sasa tool. The SASA for individual chains (LIF, gp130 and LIFR) and the entire LIF-gp130 and LIF-LIFR complex were computed separately for each snapshot at 1 ns intervals of the MD simulations. The buried surface area for the LIF-gp130 interface was calculated using the following formula:

BSA=SASA_LIF_+SASA_gp130_−SASA_complexLIF-gp130_

Similarly, the buried surface area for the LIF-LIFR interface was calculated as:

BSA=SASA_LIF_+SASA_LIFR_−SASA_complexLIF-LIFR_

This analysis was performed for each 1 ns snapshot of the MD simulations, and the average buried surface area was calculated across all snapshots to represent the interaction surfaces between LIF and its binding partners, gp130 and LIFR.

### 4.7 Clustering analysis of LIF-gp130-LIFR conformational states

To investigate the conformational states and domain interactions within the LIF-gp130-LIFR complex, we performed clustering analysis on MD simulation data using Gaussian Mixture Models (GMM). Four key features were selected: (1) the angle between LIF and the center of mass (COM) of LIFR D3 and D4, (2) the angle between LIF and the COM of gp130 D2 and D3, (3) the distance between gp130 D2 and LIFR D4, and (4) the distance between gp130 D3 and LIFR D3. The data was extracted from the MD simulations, and GMM clustering was applied to categorize the conformations into three distinct clusters, representing different conformational states of the complex. For each cluster, representative conformations were identified by selecting the structure with the minimum variance from the cluster center. And the cluster center structures were extracted from the MD simulation, and visualized in ChimeraX and Chimera[45] for structural analysis and comparison.

### 4.8 Calculation of Gibbs free energy landscape

Two features were selected for analysis: (1) the angle between LIF and the center of mass (COM) of gp130 domains D2 and D3, and (2) the angle between LIF and the COM of LIFR domains D3 and D4. Data were extracted from the MD simulations, and the GROMACS sham tool was used to calculate the Gibbs free energy landscape by Boltzmann inversion of the histogram. The Python script (https://github.com/Jerkwin/gmxtools/blob/master/xpm2all) was then employed to convert the GROMACS sham XPM file results into Excel data by adding -xyz command. The corresponding lowest energy frame structure was extracted using the GROMACS trjconv tool, and structure visualization was performed using ChimeraX.

## CRediT authorship contribution statement

**Bo Gao:** Writing – review & editing, Writing – original draft, Visualization, Methodology, Data curation, Formal analysis. **Hanrui Liu:** Writing – review & editing, Validation, Formal analysis. **Mengkai Zhu:** Methodology, Validation. **Shun Zhan:** Validation. **Meiniang Wang:** Writing – review & editing, Funding acquisition. **Yijun Rua:** Writing – review & editing, Supervision. **Yue Zheng:** Writing – review & editing, Writing – original draft, Visualization, Validation, Supervision, Project administration, Conceptualization.

## Declaration of Competing Interest

The authors declare that they have no known competing financial interests or personal relationships that could have appeared to influence the work reported in this paper.

## References

[1] Metcalf D. (1991). The leukemia inhibitory factor (LIF). Int J Cell Cloning.

[2] Nicola N.A., Babon J.J. (2015). Leukemia inhibitory factor (LIF). Cytokine Growth Factor Rev.

[3] Metcalf D., Nicola N.A., Gearing D.P. (1990). Effects of injected leukemia inhibitory factor on hematopoietic and other tissues in mice. Blood.

[4] Bauer S., Patterson P.H. (2006). Leukemia inhibitory factor promotes neural stem cell self-renewal in the adult brain. J Neurosci.

[5] Hirai H., Karian P., Kikyo N. (2011). Regulation of embryonic stem cell self-renewal and pluripotency by leukaemia inhibitory factor. Biochem J.

[6] Stewart C.L., Kaspar P., Brunet L.J., Bhatt H., Gadi I., Kontgen F., Abbondanzo S.J. (1992). Blastocyst implantation depends on maternal expression of leukaemia inhibitory factor. Nature.

[7] Charnock-Jones D.S., Sharkey A.M., Fenwick P., Smith S.K. (1994). Leukaemia inhibitory factor mRNA concentration peaks in human endometrium at the time of implantation and the blastocyst contains mRNA for the receptor at this time. J Reprod Fertil.

[8] Guo D., Dong W., Cong Y., Liu Y., Liang Y., Ye Z., Zhang J., Zhou Y. (2024). LIF Aggravates pulpitis by promoting inflammatory response in macrophages. Inflammation.

[9] Jorgensen M.M., de la Puente P. (2022). Leukemia inhibitory factor: an important cytokine in pathologies and cancer. Biomolecules.

[10] Rose-John S. (2018). Interleukin-6 family cytokines. Cold Spring Harb Perspect Biol.

[11] Kang S., Narazaki M., Metwally H., Kishimoto T. (2020). Historical overview of the interleukin-6 family cytokine. J Exp Med.

[12] Ferguson-Smith A.C., Chen Y.F., Newman M.S., May L.T., Sehgal P.B., Ruddle F.H. (1988). Regional localization of the interferon-beta 2/B-cell stimulatory factor 2/hepatocyte stimulating factor gene to human chromosome 7p15-p21. Genomics.

[13] Gearing D.P., Gough N.M., King J.A., Hilton D.J., Nicola N.A., Simpson R.J., Nice E.C., Kelso A., Metcalf D. (1987). Molecular cloning and expression of cDNA encoding a murine myeloid leukaemia inhibitory factor (LIF). EMBO J.

[14] Paul S.R., Bennett F., Calvetti J.A., Kelleher K., Wood C.R., O'Hara R.M., Leary A.C., Sibley B., Clark S.C., Williams D.A. (1990). Molecular cloning of a cDNA encoding interleukin 11, a stromal cell-derived lymphopoietic and hematopoietic cytokine. Proc Natl Acad Sci USA.

[15] Bazan J.F. (1991). Neuropoietic cytokines in the hematopoietic fold. Neuron.

[16] Rose T.M., Bruce A.G. (1991). Oncostatin M is a member of a cytokine family that includes leukemia-inhibitory factor, granulocyte colony-stimulating factor, and interleukin 6. Proc Natl Acad Sci USA.

[17] Pennica D., King K.L., Shaw K.J., Luis E., Rullamas J., Luoh S.M., Darbonne W.C., Knutzon D.S., Yen R., Chien K.R. (1995). Expression cloning of cardiotrophin 1, a cytokine that induces cardiac myocyte hypertrophy. Proc Natl Acad Sci USA.

[18] Senaldi G., Varnum B.C., Sarmiento U., Starnes C., Lile J., Scully S., Guo J., Elliott G., McNinch J., Shaklee C.L., Freeman D., Manu F., Simonet W.S., Boone T., Chang M.S. (1999). Novel neurotrophin-1/B cell-stimulating factor-3: a cytokine of the IL-6 family. Proc Natl Acad Sci USA.

[19] Smith A.G., Heath J.K., Donaldson D.D., Wong G.G., Moreau J., Stahl M., Rogers D. (1988). Inhibition of pluripotential embryonic stem cell differentiation by purified polypeptides. Nature.

[20] Hilton D.J., Nicola N.A., Metcalf D. (1991). Distribution and comparison of receptors for leukemia inhibitory factor on murine hemopoietic and hepatic cells. J Cell Physiol.

[21] Austin L., Burgess A.W. (1991). Stimulation of myoblast proliferation in culture by leukaemia inhibitory factor and other cytokines. J Neurol Sci.

[22] Deverman B.E., Patterson P.H. (2012). Exogenous leukemia inhibitory factor stimulates oligodendrocyte progenitor cell proliferation and enhances hippocampal remyelination. J Neurosci.

[23] Gough N.M., Williams R.L., Hilton D.J., Pease S., Willson T.A., Stahl J., Gearing D.P., Nicola N.A., Metcalf D. (1989). LIF: a molecule with divergent actions on myeloid leukaemic cells and embryonic stem cells. Reprod Fertil Dev.

[24] Wulansari N., Sulistio Y.A., Darsono W.H.W., Kim C.H., Lee S.H. (2021). LIF maintains mouse embryonic stem cells pluripotency by modulating TET1 and JMJD2 activity in a JAK2-dependent manner. Stem Cells.

[25] Boulanger M.J., Bankovich A.J., Kortemme T., Baker D., Garcia K.C. (2003). Convergent mechanisms for recognition of divergent cytokines by the shared signaling receptor gp130. Mol Cell.

[26] Metcalfe R.D., Putoczki T.L., Griffin M.D.W. (2020). Structural Understanding of Interleukin 6 Family Cytokine Signaling and Targeted Therapies: Focus on Interleukin 11. Front Immunol.

[27] Garbers C., Aparicio-Siegmund S., Rose-John S. (2015). The IL-6/gp130/STAT3 signaling axis: recent advances towards specific inhibition. Curr Opin Immunol.

[28] Zhang J.G., Owczarek C.M., Ward L.D., Howlett G.J., Fabri L.J., Roberts B.A., Nicola N.A. (1997). Evidence for the formation of a heterotrimeric complex of leukaemia inhibitory factor with its receptor subunits in solution. Biochem J.

[29] Zhou Y., Stevis P.E., Cao J., Saotome K., Wu J., Glatman Zaretsky A., Haxhinasto S., Yancopoulos G.D., Murphy A.J., Sleeman M.W., Olson W.C., Franklin M.C. (2023). Structural insights into the assembly of gp130 family cytokine signaling complexes. Sci Adv.

[30] Hinds M.G., Maurer T., Zhang J.G., Nicola N.A., Norton R.S. (1998). Solution structure of leukemia inhibitory factor. J Biol Chem.

[31] Robinson R.C., Grey L.M., Staunton D., Vankelecom H., Vernallis A.B., Moreau J.F., Stuart D.I., Heath J.K., Jones E.Y. (1994). The crystal structure and biological function of leukemia inhibitory factor: implications for receptor binding. Cell.

[32] Huyton T., Zhang J.G., Luo C.S., Lou M.Z., Hilton D.J., Nicola N.A., Garrett T.P. (2007). An unusual cytokine:Ig-domain interaction revealed in the crystal structure of leukemia inhibitory factor (LIF) in complex with the LIF receptor. Proc Natl Acad Sci USA.

[33] Jin M., Seed R.I., Cai G., Shing T., Wang L., Ito S., Cormier A., Wankowicz S.A., Jespersen J.M., Baron J.L., Carey N.D., Campbell M.G., Yu Z., Tang P.K., Cossio P., Wen W., Lou J., Marks J., Nishimura S.L., Cheng Y. (2024). Dynamic allostery drives autocrine and paracrine TGF-beta signaling. Cell.

[34] Huang J., Rauscher S., Nawrocki G., Ran T., Feig M., de Groot B.L., Grubmuller H., MacKerell A.D., Jr (2017). CHARMM36m: an improved force field for folded and intrinsically disordered proteins. Nat Methods.

[35] Hess B., Kutzner C., van der Spoel D., Lindahl E. (2008). GROMACS 4: Algorithms for Highly Efficient, Load-Balanced, and Scalable Molecular Simulation. J Chem Theory Comput.

[36] Plun-Favreau H., Perret D., Diveu C., Froger J., Chevalier S., Lelievre E., Gascan H., Chabbert M. (2003). Leukemia inhibitory factor (LIF), cardiotrophin-1, and oncostatin M share structural binding determinants in the immunoglobulin-like domain of LIF receptor. J Biol Chem.

[37] Hudson K.R., Vernallis A.B., Heath J.K. (1996). Characterization of the receptor binding sites of human leukemia inhibitory factor and creation of antagonists. J Biol Chem.

[38] Davis A.M., St-Gallay S.A., Kleywegt G.J. (2008). Limitations and lessons in the use of X-ray structural information in drug design. Drug Discov Today.

[39] Renaud J.P., Chari A., Ciferri C., Liu W.T., Remigy H.W., Stark H., Wiesmann C. (2018). Cryo-EM in drug discovery: achievements, limitations and prospects. Nat Rev Drug Discov.

[40] Sims N.A., Johnson R.W. (2012). Leukemia inhibitory factor: a paracrine mediator of bone metabolism. Growth Factors.

[41] Gadient R.A., Patterson P.H. (1999). Leukemia inhibitory factor, Interleukin 6, and other cytokines using the GP130 transducing receptor: roles in inflammation and injury. Stem Cells.

[42] Humphrey W., Dalke A., Schulten K. (1996). VMD: visual molecular dynamics. J Mol Graph.

[43] Meng E.C., Goddard T.D., Pettersen E.F., Couch G.S., Pearson Z.J., Morris J.H., Ferrin T.E. (2023). UCSF ChimeraX: tools for structure building and analysis. Protein Sci.

[44] Grant B.J., Rodrigues A.P., ElSawy K.M., McCammon J.A., Caves L.S. (2006). Bio3d: an R package for the comparative analysis of protein structures. Bioinformatics.

[45] Pettersen E.F., Goddard T.D., Huang C.C., Couch G.S., Greenblatt D.M., Meng E.C., Ferrin T.E. (2004). UCSF Chimera--a visualization system for exploratory research and analysis. J Comput Chem.

