# Supplementary material for: Molecular dynamics simulations reveal key roles of the LIF receptor in the assembly of human LIF signaling complex
Source: Comput Struct Biotechnol J. 2025 Jan 27;27:585–94. doi: 10.1016/j.csbj.2025.01.014 (PMC11847480; doi:10.1016/j.csbj.2025.01.014)
Supplement: Supplementary file 3 — Supplementary material [file mmc1.docx]

**Supporting Information**


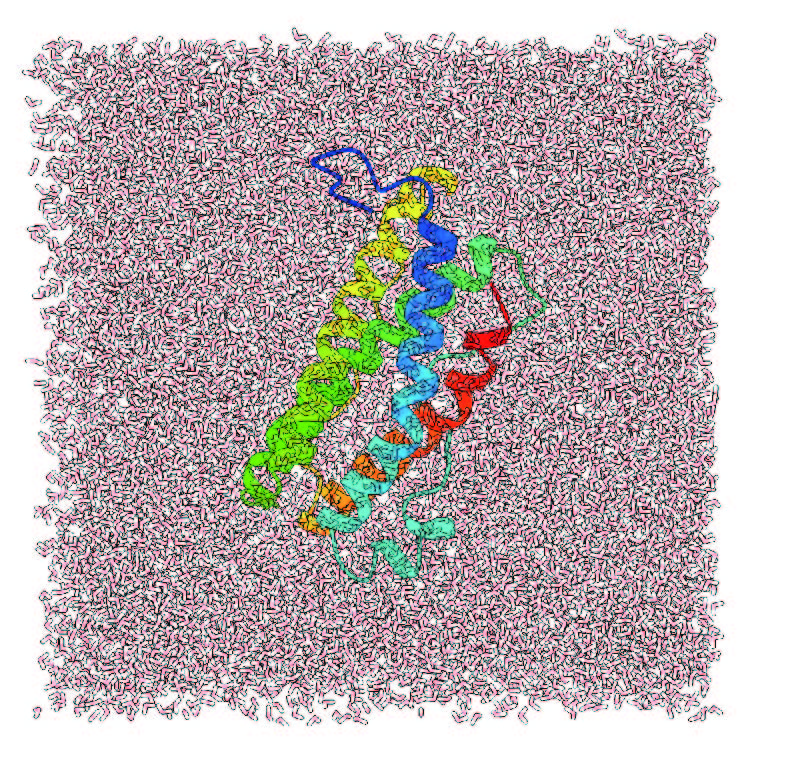


**Fig. S1 Snapshot of molecular dynamics (MD) simulation of the LIF solution system.**

Water molecules are shown as red and white sticks, and the LIF structure is depicted in cartoon style with a rainbow color scheme.

**
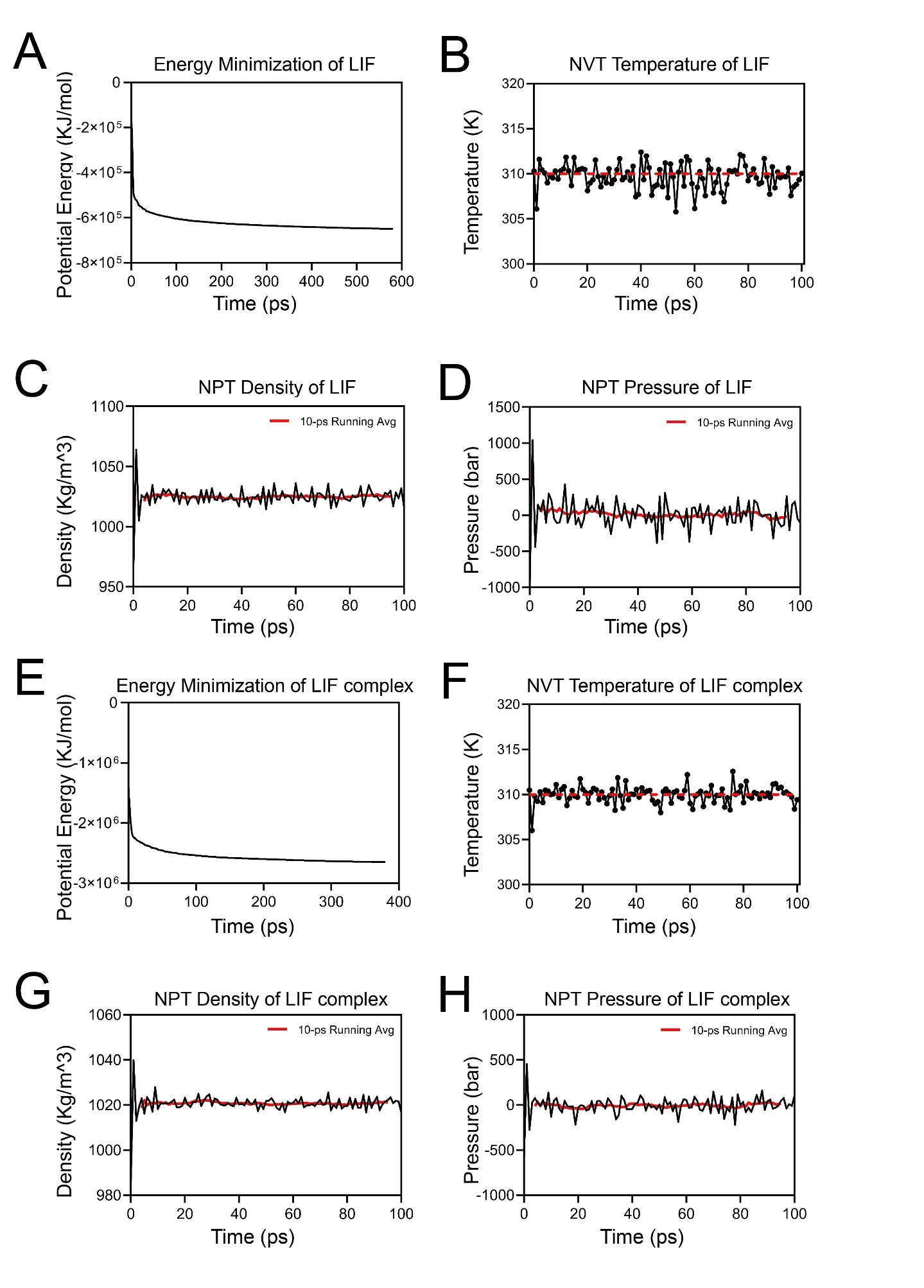
**

**Fig. S2 Molecular dynamics simulations of the LIF and LIF core complex system.**

(A) Potential energy of the LIF system during energy minimization.

(B) NVT temperature of the LIF system. The red line represents the target system temperature of 310K.

(C) NPT density of the LIF system. The red line represents the 10-ps running average of the density.

(D) NPT pressure of the LIF system. The red line represents the 10-ps running average of the pressure.

(E) Potential energy of the LIF core complex system during energy minimization.

(F) NVT temperature of the LIF core complex system. The red line represents the target system temperature of 310K.

(G) NPT density of the LIF core complex system. The red line represents the 10-ps running average of the density.

(H) NPT pressure of the LIF core complex system. The red line represents the 10-ps running average of the pressure.


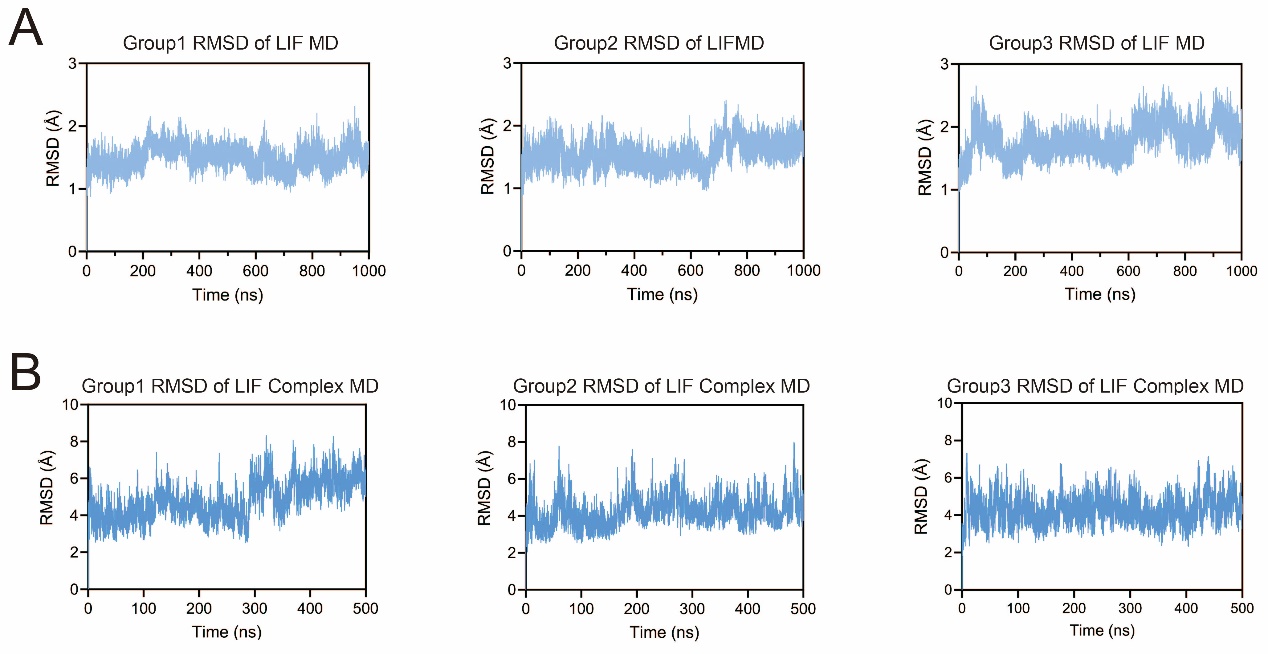


**Fig. S3 Root mean square deviations (RMSD) of the three independent molecular dynamics simulation groups.**

(A) RMSD of the three independent LIF MD simulation groups.

(B) RMSD of the three independent LIF core complex MD simulation groups.


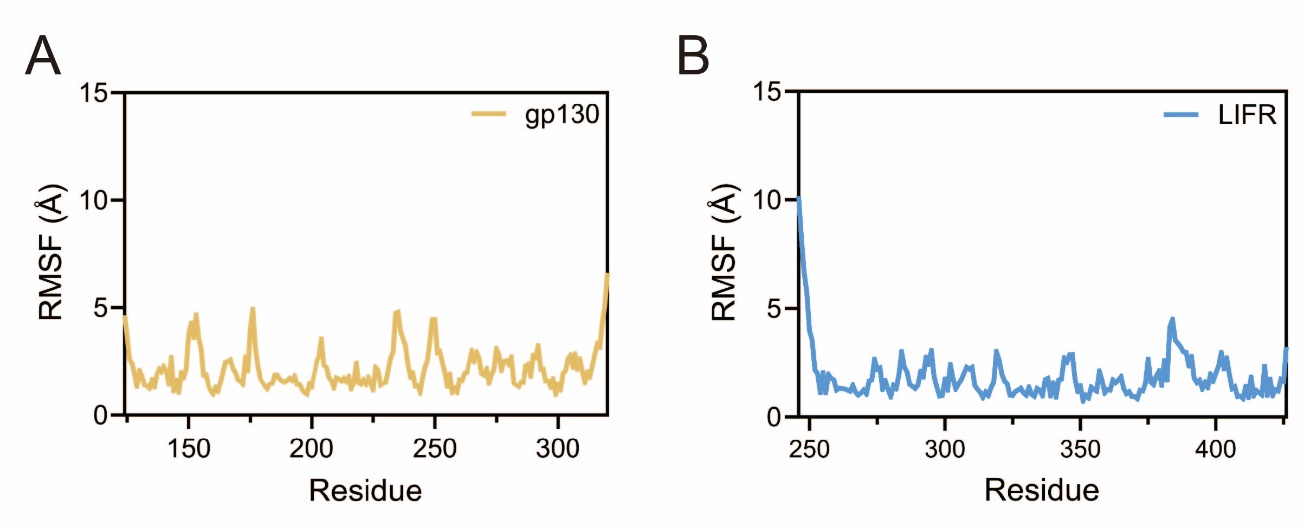


**Fig. S4 Root mean square fluctuation (RMSF) of the gp130 and LIFR subunits in the LIF core complex MD simulation.**

(A) RMSF of the gp130 subunit in the LIF core complex MD simulation.

(B) RMSF of the LIFR subunit in the LIF core complex MD simulation.


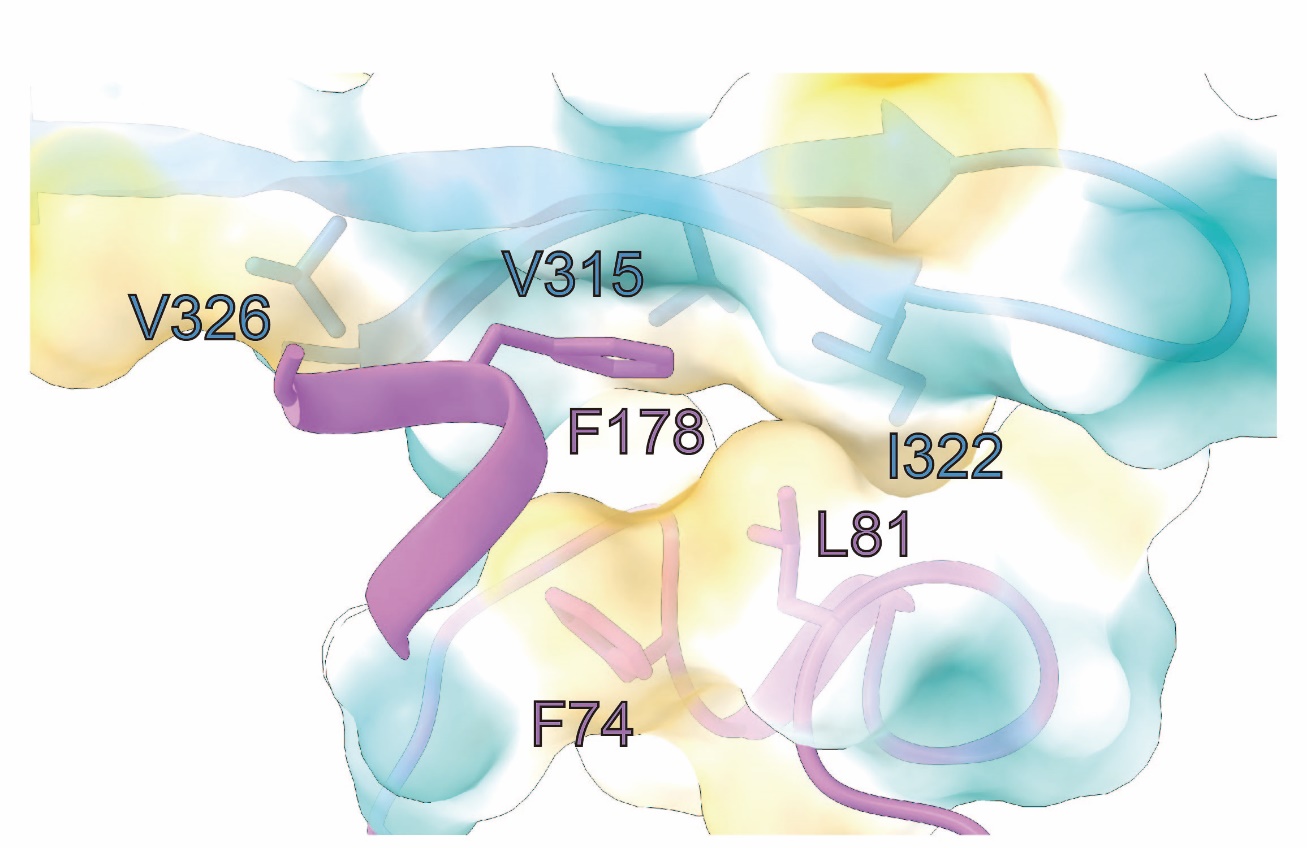


**Fig. S5 F178 of LIF binds into a hydrophobic pocket formed by the D3 domain of LIFR and the AB loop turn of LIF.**

The surfaces of LIFR and the AB loop turn of LIF are colored according to hydrophobic potential. The surface coloring ranges from dark goldenrod for the most hydrophobic potentials to dark cyan for the most hydrophilic potentials.


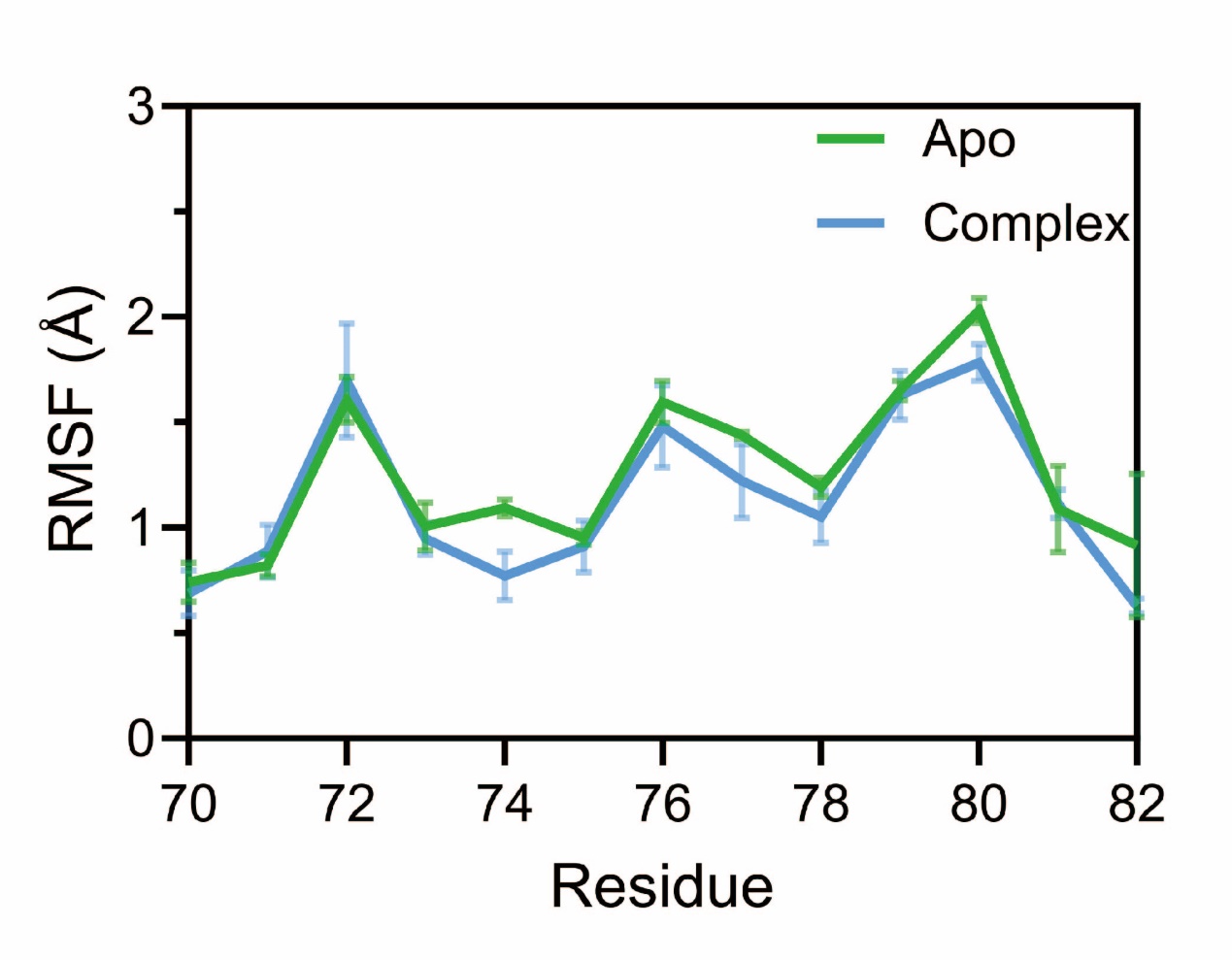


**Fig. S6 RMSF of the AB loop turn in the LIF and LIF core complex MD simulation.**

The green line represents the apo system, and the blue line represents the complex system. Error bars represent mean ± SD of n = 3 experiments.


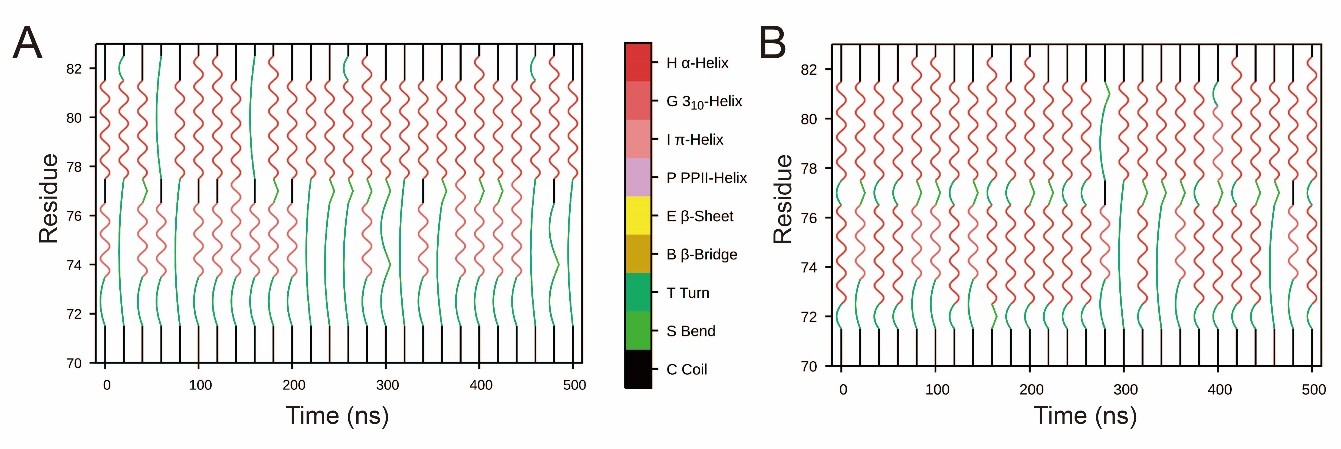


**Fig. S7** **Characteristics of the AB loop turn’s secondary structure.**

(A) Secondary structure characteristics of the AB loop turn in the LIF MD simulation.

(B) Secondary structure characteristics of the AB loop turn in the LIF core complex MD simulation.


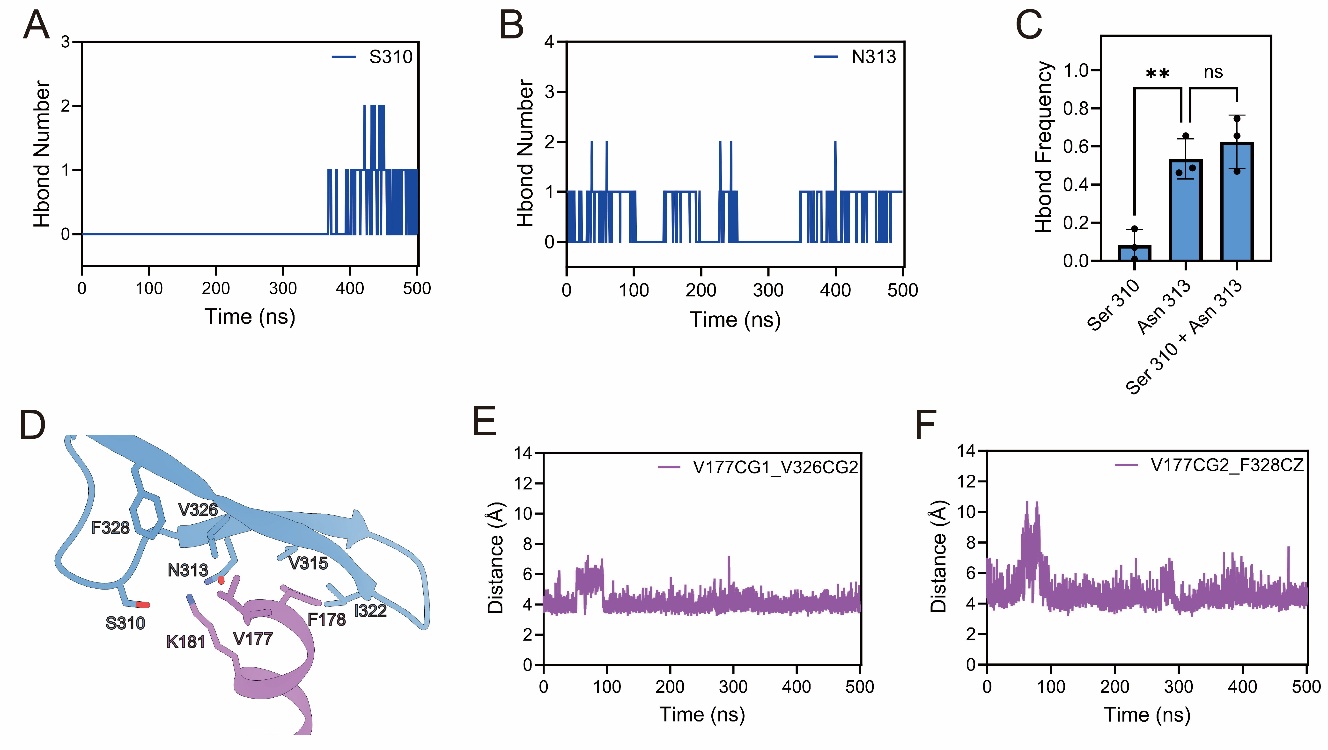
**Fig. S8 K181 forms hydrogen bonds with S310 and N313 of LIFR.**

(A) K181 forms hydrogen bond with S310 during the MD simulation.

(B) K181 forms hydrogen bond with N313 during the MD simulation.

(C) Hydrogen bond frequency statistics of K181 interactions with S310 and N313. Error bars represent mean ± SD of n = 3 experiments. **, p < 0.01. ns, P > 0.05.

(D) The β-hairpin motif brings the loop closer to K181.

(E) The distance between the CG1 atom of V177 in LIF and the CG2 atom of V326 in LIFR during the MD simulation.

(F) The distance between the CG2 atom of V177 in LIF and the CZ atom of F328 in LIFR during the MD simulation.


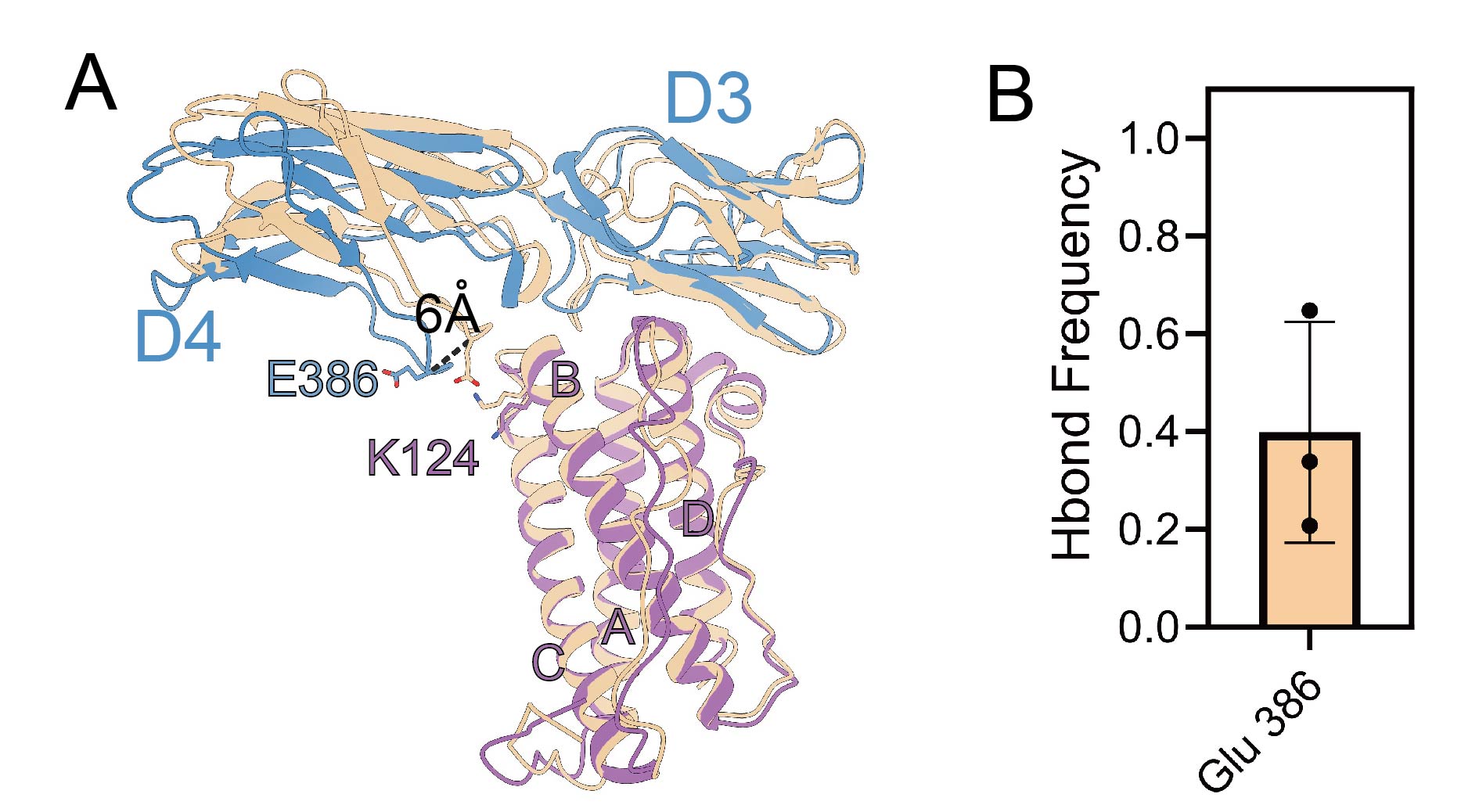


**Fig. S9 K124 undergoes an upward flip triggering electrostatic interaction and hydrogen bond formation.**

(A) Overall structural view showing K124 undergoing an upward flip, positioned to interaction with E386. The loop in the D4 domain displays a 6 Å inward displacement relative to LIF. The representative structure of the K124-E386 electrostatic interaction and hydrogen bond formation is shown in vanilla-colored cartoon style. The cryo-EM structure of LIF is shown in purple, and the LIFR is displayed in blue.

(B) Hydrogen bond frequency statistics of K124 interaction with E386. Error bars represent mean ± SD of n = 3 experiments.


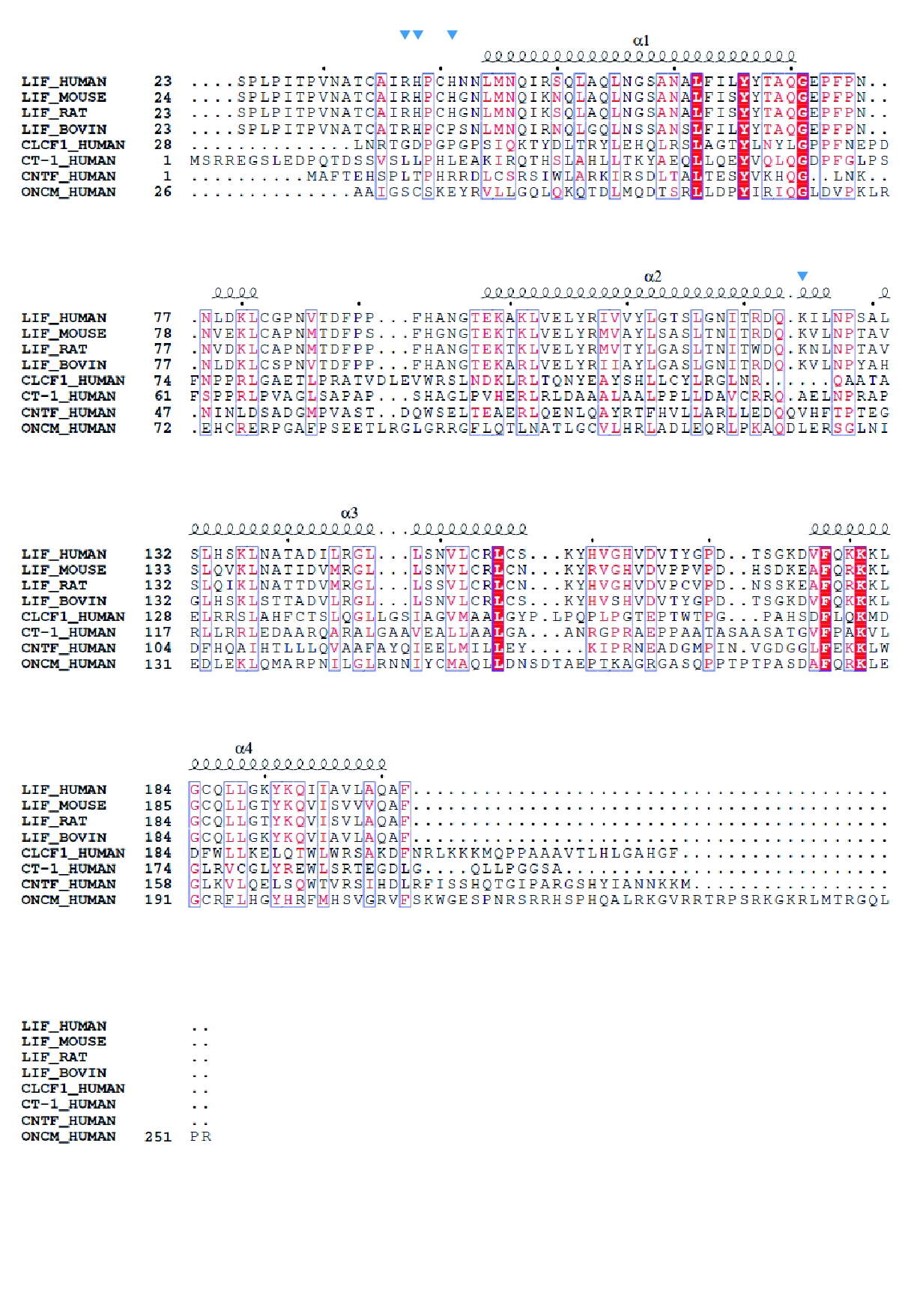


**Fig. S10 Sequence alignment of LIF homologs.**

The sequences were aligned using Clustal Omega and the figure was prepared using ESPript3.0. Cyan triangles indicate residues making electrostatic interactions.


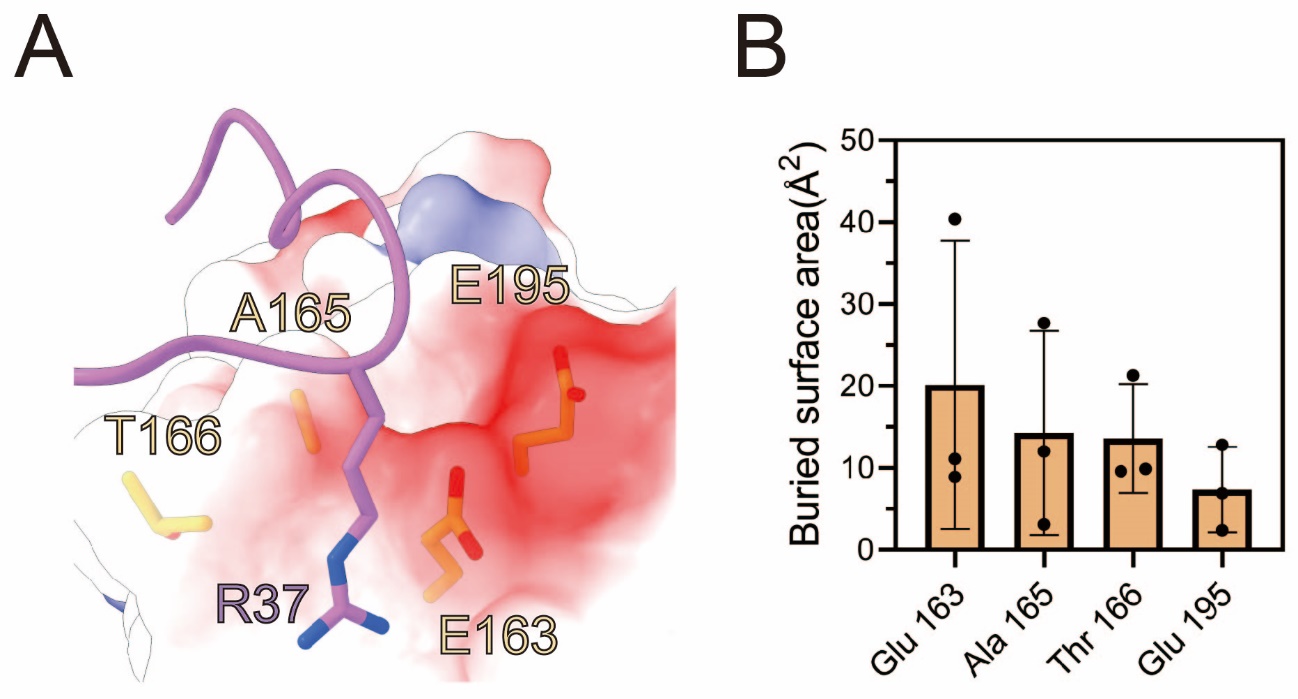


**Fig. S11 R37 makes electrostatic interactions with gp130 during the MD simulation.**

(A) R37 is positioned to make electrostatic interactions with gp130. Contact residues are shown as sticks. gp130 surface is colored according to the electrostatic surface potential (blue, +5 kT; red, −5 kT).

(B) The representative residues of gp130 interact with R37 during the MD simulation. Error bars represent mean ± SD of n = 3 experiments.


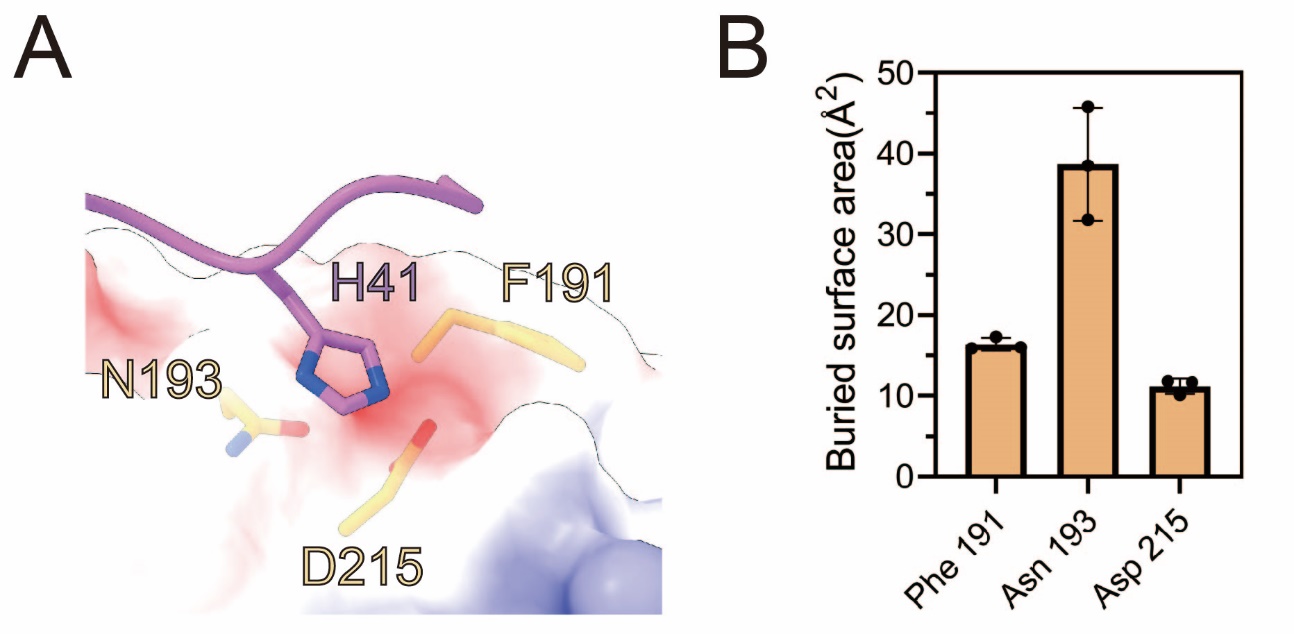


**Fig. S12 H41 makes electrostatic interactions with gp130 during the MD simulation.**

(A) H41 is positioned to make electrostatic interactions with gp130. Contact residues are shown as sticks. gp130 surface is colored according to the electrostatic surface potential (blue, +5 kT; red, −5 kT).

(B) The representative residues of gp130 interact with H41 during the MD simulation. Error bars represent mean ± SD of n = 3 experiments.


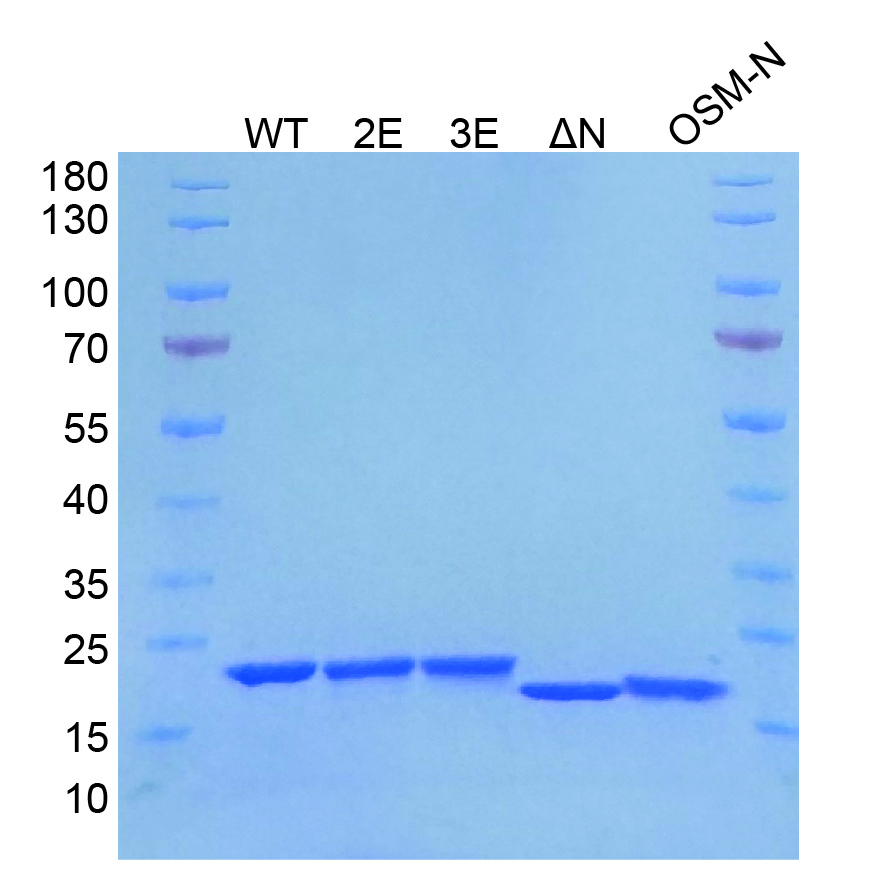


**Fig. S13 SDS–PAGE of WT LIF and its variants.**


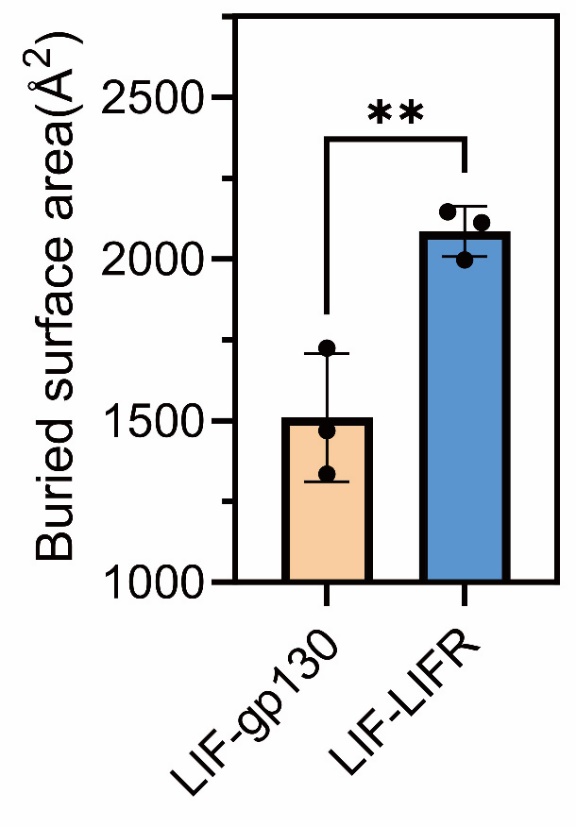


**Fig. S14 Buried surface area of LIF-LIFR and LIF-gp130 Interfaces.**

Error bars represent mean ± SD of n = 3 experiments. **, p < 0.01.


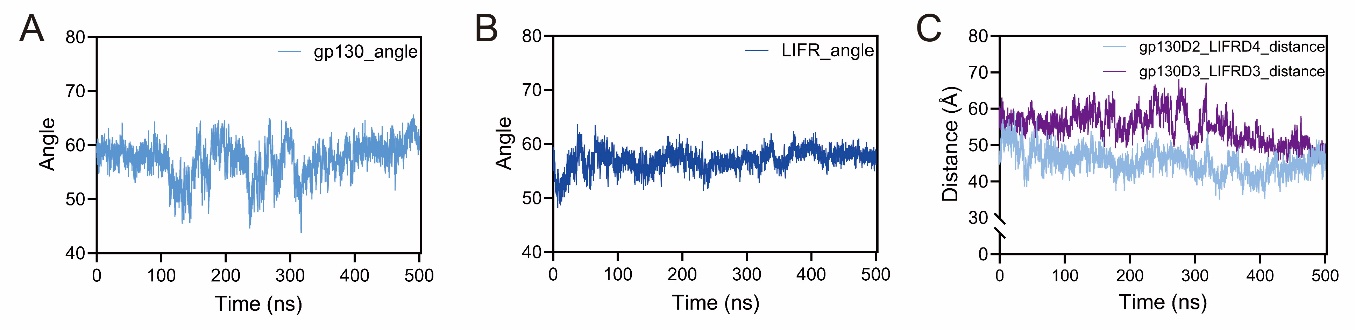


**Fig. S15 Statistics of gp130-LIF-LIFR core complex MD simulation**

(A) The angle of between LIF and the center of mass (COM) of the gp130 D2 and D3 domains during the MD simulation.

(B) The angle of between LIF and the COM of the LIFR D3 and D4 domains during the MD simulation.

(C) Distances between gp130 and LIFR during the MD simulation. The distance between gp130 D2 and LIFR D4 is shown by the purple line, while the distance between gp130 D3 and LIFR D3 is represented by the sky-blue line.


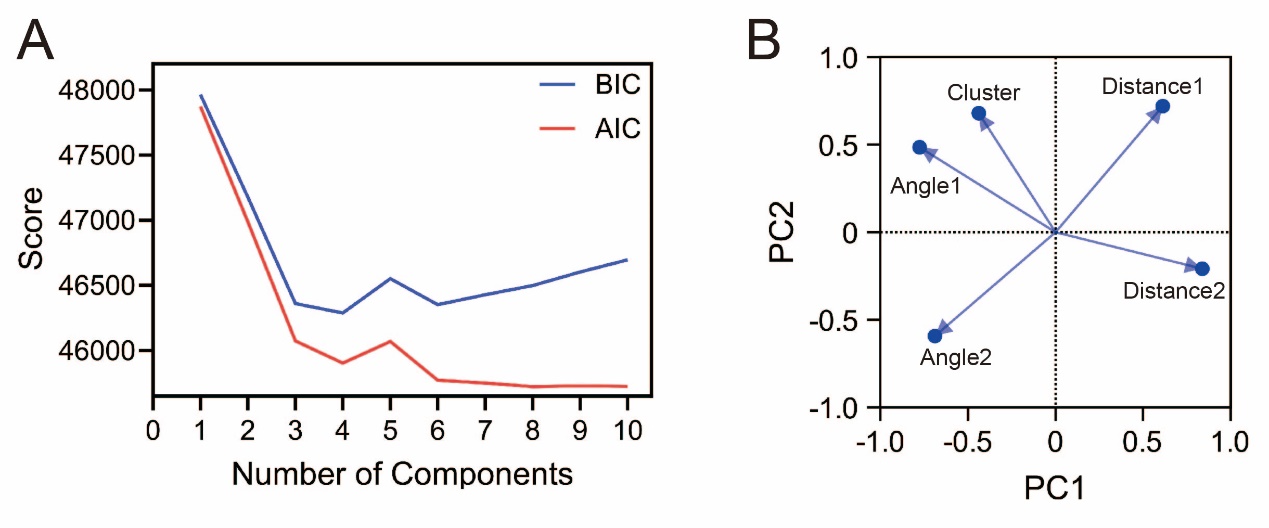


**Fig. S16 Statistical data processing.**

(A) Bayesian Information Criterion (BIC) and Akaike Information Criterion (AIC) curve scores of the Elbow classification.

(B) Principal component analysis (PCA) loadings of the dataset with five features. "Angle1" represents the angle between LIF and the COM of the gp130 D2 and D3, "Angle2" represents the angle between LIF and the COM of the LIFR D3 and D4, "Distance1" represents the distance between gp130 D2 and LIFR D4, and "Distance2" represents while the distance between gp130 D3 and LIFR D3.


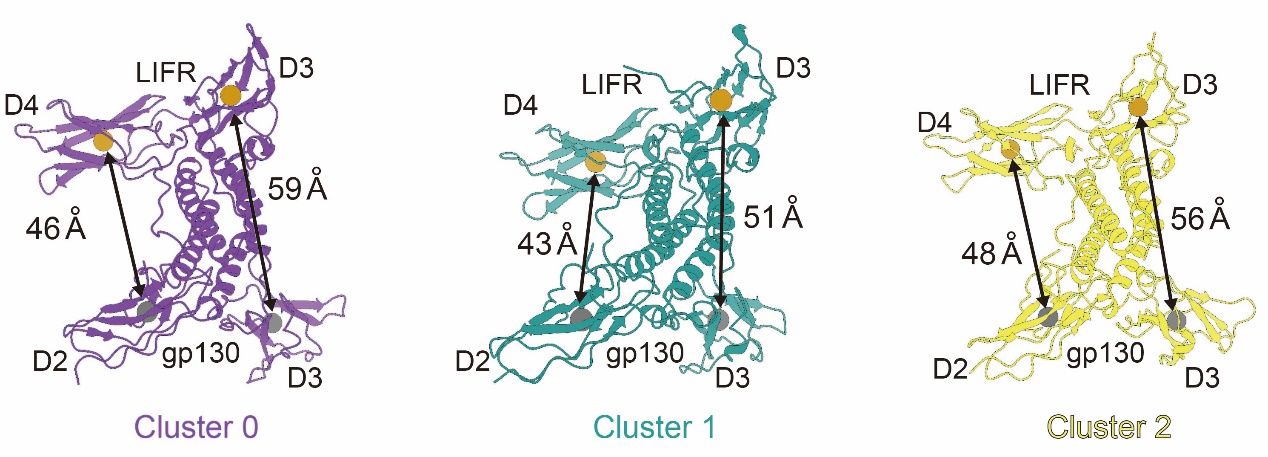


**Fig. S17 Distances between gp130 and LIFR in the representative structures of each cluster.**

The representative structure of Cluster 0 is shown in purple, Cluster 1 in teal, and Cluster 2 in yellow. The dark goldenrod spheres represent the COM of each domain of LIFR, and the dim gray spheres represent the COM of each domain of gp130. The distances between gp130 and LIFR are labeled for each cluster. The yellow ball represents the COM of the LIFR domains, and the gray ball represents COM of the gp130 domains.


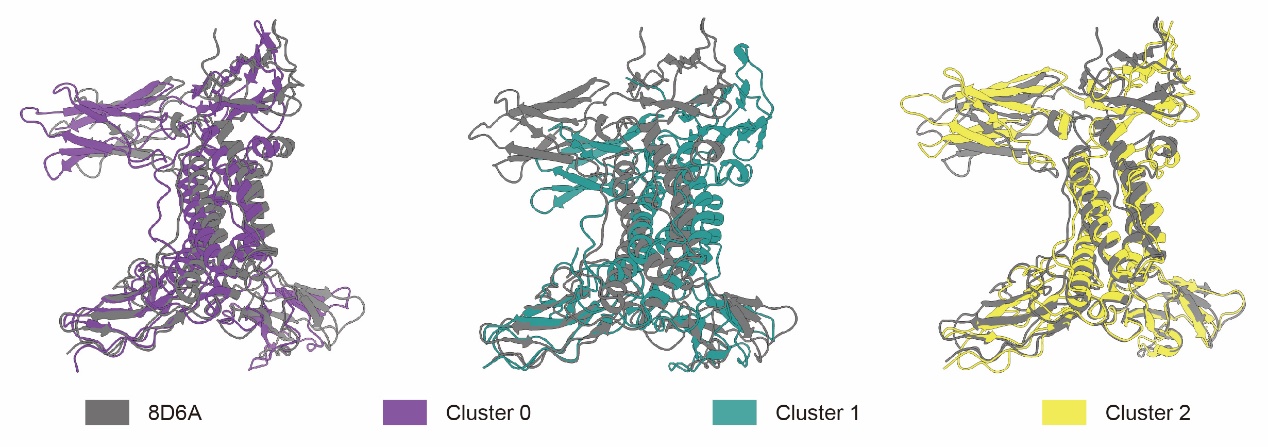


**Fig. S18** **Superposition of the representative structures of each cluster and the cryo-EM structure.**

The representative structure of Cluster 0 is shown in purple, Cluster 1 in teal, Cluster 2 in yellow, and the cryo-EM structure (PDB: 8D6A) is shown in gray.


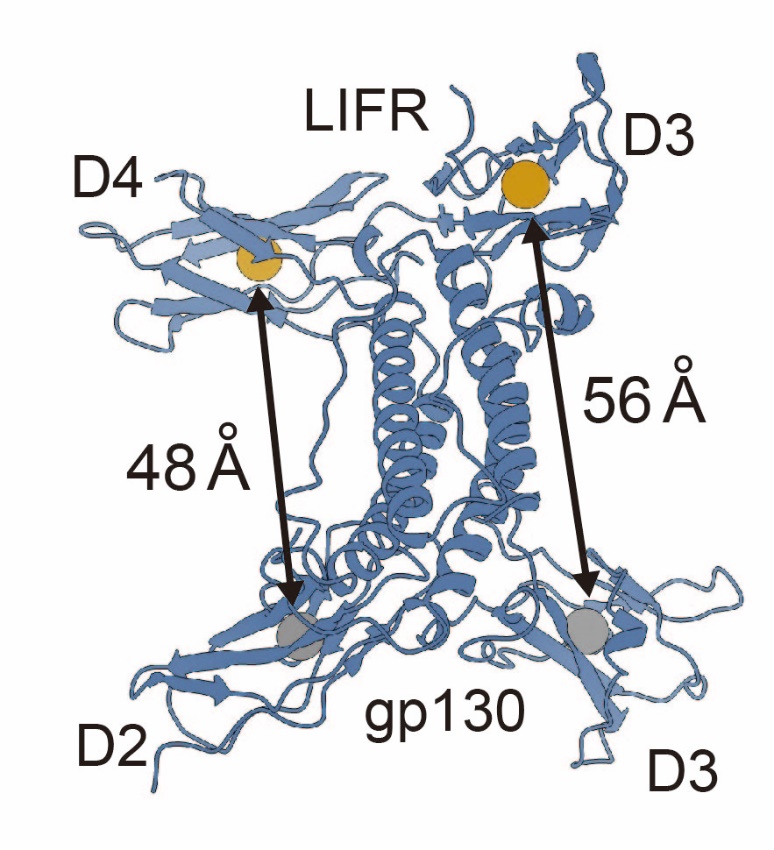


**Fig. S19 Distances between gp130 and LIFR in the structure of** **Gibbs free energy landscape minimum.**

The structure corresponding to the minimum of the Gibbs free energy landscape is displayed in gray-blue. The yellow ball represents the COM of the LIFR domains, and the dim gray ball represents COM of the gp130 domains.


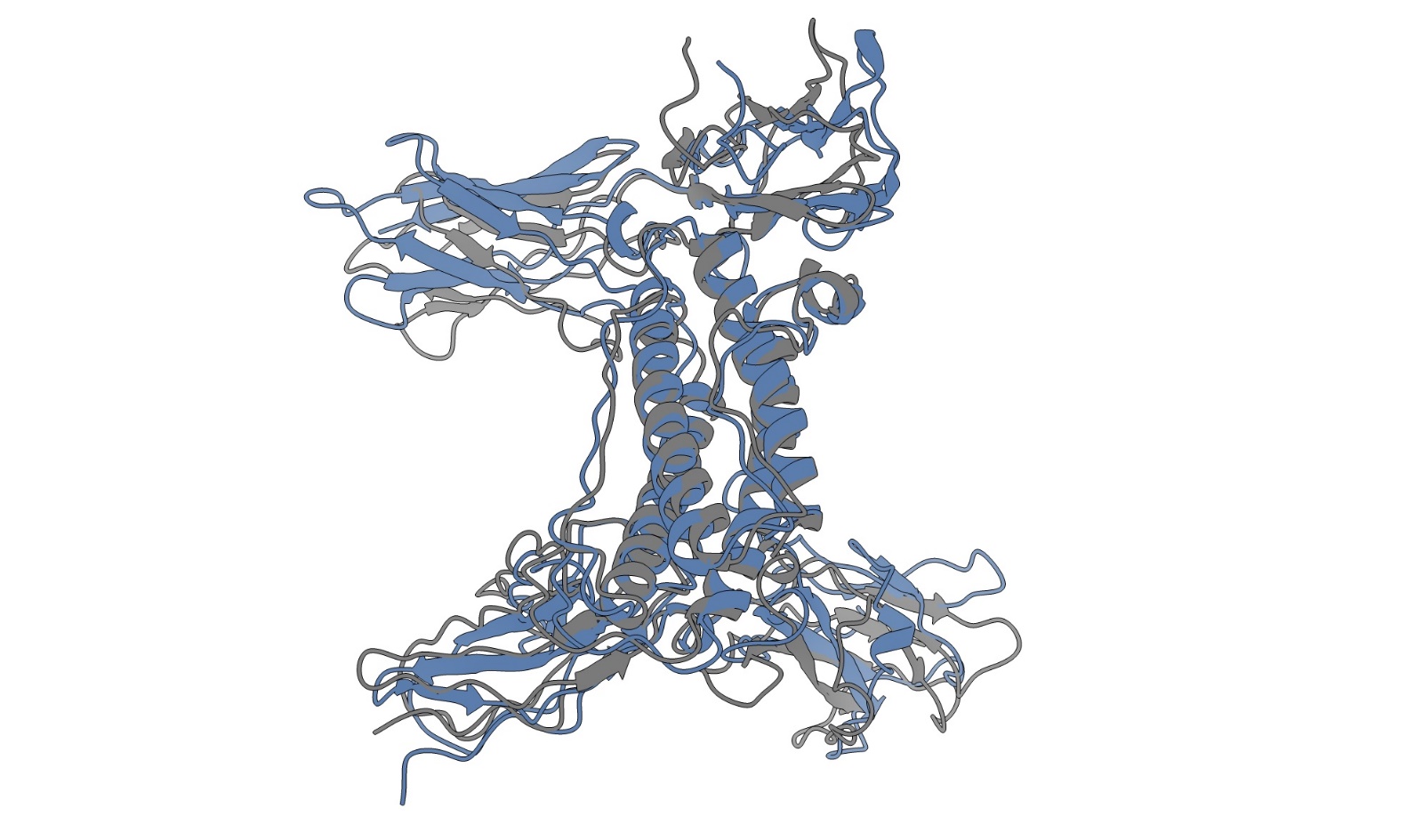


**Fig. S20 Superposition of the structure of Gibbs free energy landscape minimum and the cryo-EM structure.**

The structure corresponding to the minimum of the Gibbs free energy landscape is displayed in gray-blue, and the cryo-EM structure is shown in gray.

**Video 1 LIF MD simulation over 1 μs.**

One frame was captured every 2 ns. LIF is shown in cartoon style with rainbow color scheme.

**Video 2 LIF core complex MD simulation over 500 ns.**

One frame was captured every 1 ns. LIF is shown as purple color, gp130 is colored in goldenrod, and LIFR is displayed in blue.
